# Supplementary material for: A task-invariant prior explains trial-by-trial active avoidance behaviour across gain and loss tasks
Source: Commun Psychol. 2025 May 22;3:82. doi: 10.1038/s44271-025-00254-1 (PMC12098998; doi:10.1038/s44271-025-00254-1)
Supplement: Supplementary file 2 — Supplementary Information [file 44271_2025_254_MOESM2_ESM.pdf]

Supplementary Note 1
Supplementary Results
Model agnostic results

Regression model 1, success and value. Below you find the full results from regression model 1. The model was fitted as a cross-classified Bayesian logistic regression model specified as follows:

Choice ~ Intercept + (offer-cost) * last trial success +
(Intercept + (offer-cost) * last trial success| subject) +
(Intercept + (offer-cost) * last trial success| stimulus)

Table S1. Robber task block 1, model 1: population-level effects

	Estimate	Est.Error	l-95% CI	u-95% CI	BF_{10}
Intercept	-1.242	0.093	-1.428	-1.060	-
Success Last	0.031	0.109	-0.185	0.242	0.114
Offer - Cost	0.048	0.002	0.043	0.053	9.476e+19
Success x Offer-Cost	0.023	0.004	0.016	0.031	6.675e+14

Table S2. Robber task block 1, model 1: group-level effects

Group		Estimate	Est.Error	l-95% CI	u-95% CI
Subject SD	Intercept	1.275	0.077	1.130	1.431
	Success Last	0.763	0.122	0.525	1.001
	Offer - Cost	0.030	0.002	0.027	0.034
	Success x Offer-Cost	0.028	0.003	0.022	0.034
Subject Corr	Intercept, Success Last	0.214	0.139	-0.054	0.486
	Intercept, Offer-Cost	-0.637	0.048	-0.726	-0.535
	Success Last, Offer-Cost	-0.180	0.132	-0.438	0.075
	Intercept, Success Last x Offer-Cost	0.203	0.102	-0.002	0.394
	Success Last, Success Last x Offer-Cost	-0.557	0.111	-0.745	-0.313
	Offer-Cost, Success Last x Offer-Cost	-0.200	0.112	-0.409	0.028
Stimulus SD	Intercept	0.117	0.053	0.013	0.222
	Success Last	0.157	0.094	0.009	0.358
	Offer - Cost	0.003	0.001	0.002	0.005
	Success x Offer-Cost	0.007	0.002	0.002	0.012
Stimulus Corr	Intercept, Success Last	-0.167	0.326	-0.721	0.520
	Intercept, Offer-Cost	-0.427	0.273	-0.833	0.219
	Success Last, Offer-Cost	0.025	0.307	-0.580	0.599
	Intercept, Success Last x Offer-Cost	-0.093	0.295	-0.643	0.491
	Success Last, Success Last x Offer-Cost	-0.382	0.318	-0.847	0.363
	Offer-Cost, Success Last x Offer-Cost	-0.120	0.262	-0.595	0.419

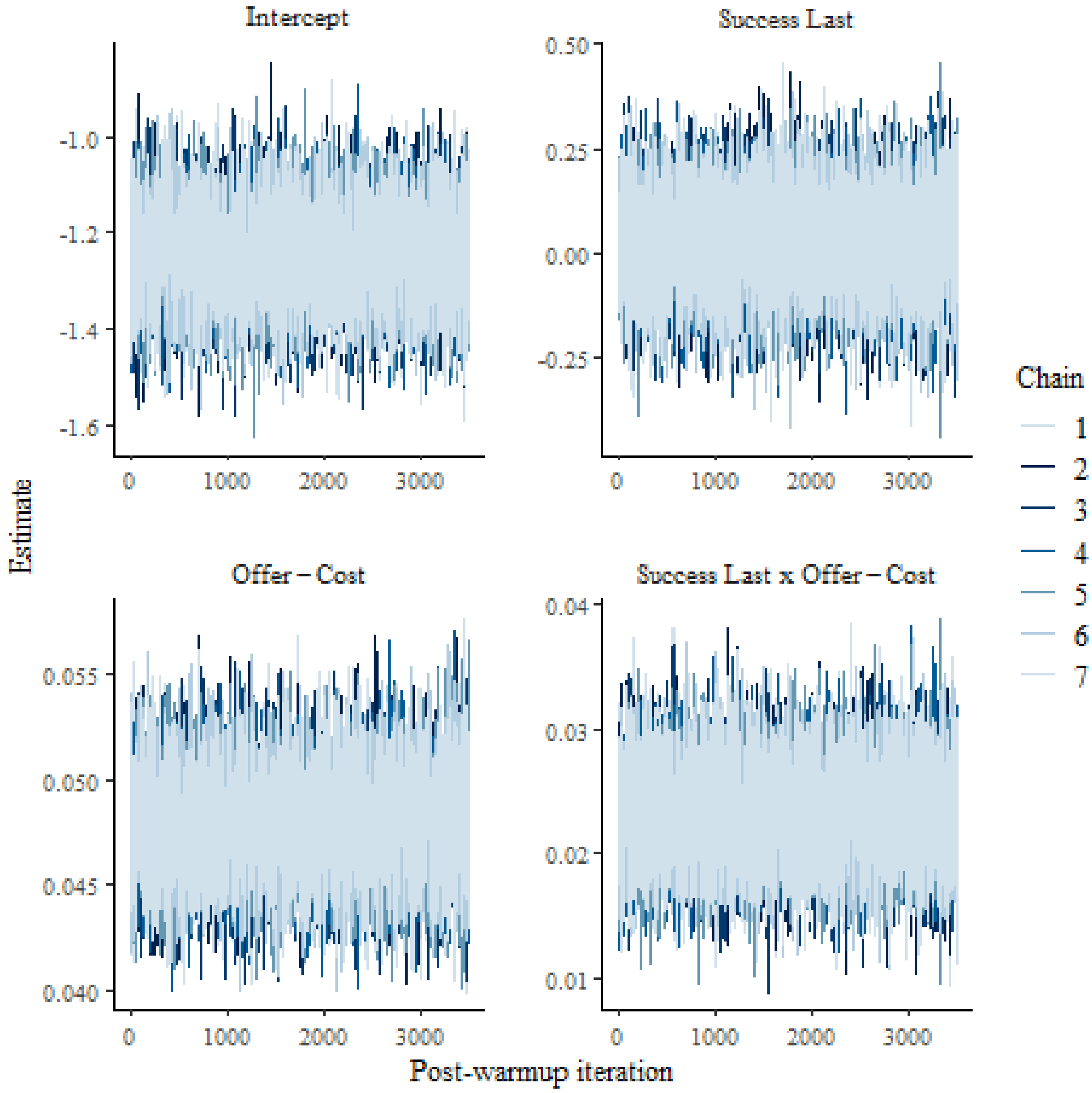

Figure S1. Trace plots for the population-level effects in model 1 fit in the first block of the robber task. *The plot is a diagnostic plot for Markov chain Monte Carlo (MCMC) estimation showing each draw for the iterations of each chain. The model converged with Rhat = 1 for all estimates except for the correlation between the group-level success on the last trial for subjects and the group-level offer-cost effect, here Rhat = 1.01.*

Table S3. Robber task block 2, model 1: population-level effects

	Estimate	Est.Error	l-95% CI	u-95% CI	BF_{10}
Intercept	-1.150	0.089	-1.324	-0.979	-
Success Last	0.199	0.106	-0.015	0.401	0.597
Offer - Cost	0.043	0.002	0.039	0.048	2.18e+14
Success x Offer-Cost	0.031	0.004	0.023	0.038	1.652e+15

Table S4. Robber task block 2, model 1: group-level effects

Group		Estimate	Est.Error	l-95% CI	u-95% CI
Subject SD	Intercept	1.254	0.075	1.114	1.407
	Success Last	0.812	0.123	0.577	1.057
	Offer - Cost	0.029	0.002	0.025	0.032
	Success x Offer-Cost	0.030	0.003	0.024	0.037
Subject Corr	Intercept, Success Last	0.603	0.112	0.366	0.804
	Intercept, Offer-Cost	-0.549	0.056	-0.651	-0.432
	Success Last, Offer-Cost	-0.416	0.119	-0.640	-0.175
	Intercept, Success Last x Offer-Cost	0.017	0.103	-0.189	0.217
	Success Last, Success Last x Offer-Cost	-0.546	0.104	-0.727	-0.323
	Offer-Cost, Success Last x Offer-Cost	0.046	0.115	-0.176	0.273
Stimulus SD	Intercept	0.030	0.024	0.001	0.088
	Success Last	0.101	0.071	0.004	0.262
	Offer - Cost	0.001	0.000	0.000	0.002
	Success x Offer-Cost	0.003	0.002	0.000	0.007
Stimulus Corr	Intercept, Success Last	-0.024	0.332	-0.648	0.613
	Intercept, Offer-Cost	-0.072	0.338	-0.684	0.594
	Success Last, Offer-Cost	-0.050	0.331	-0.659	0.596
	Intercept, Success Last x Offer-Cost	-0.037	0.332	-0.654	0.603
	Success Last, Success Last x Offer-Cost	-0.052	0.332	-0.660	0.594
	Offer-Cost, Success Last x Offer-Cost	-0.112	0.335	-0.707	0.563

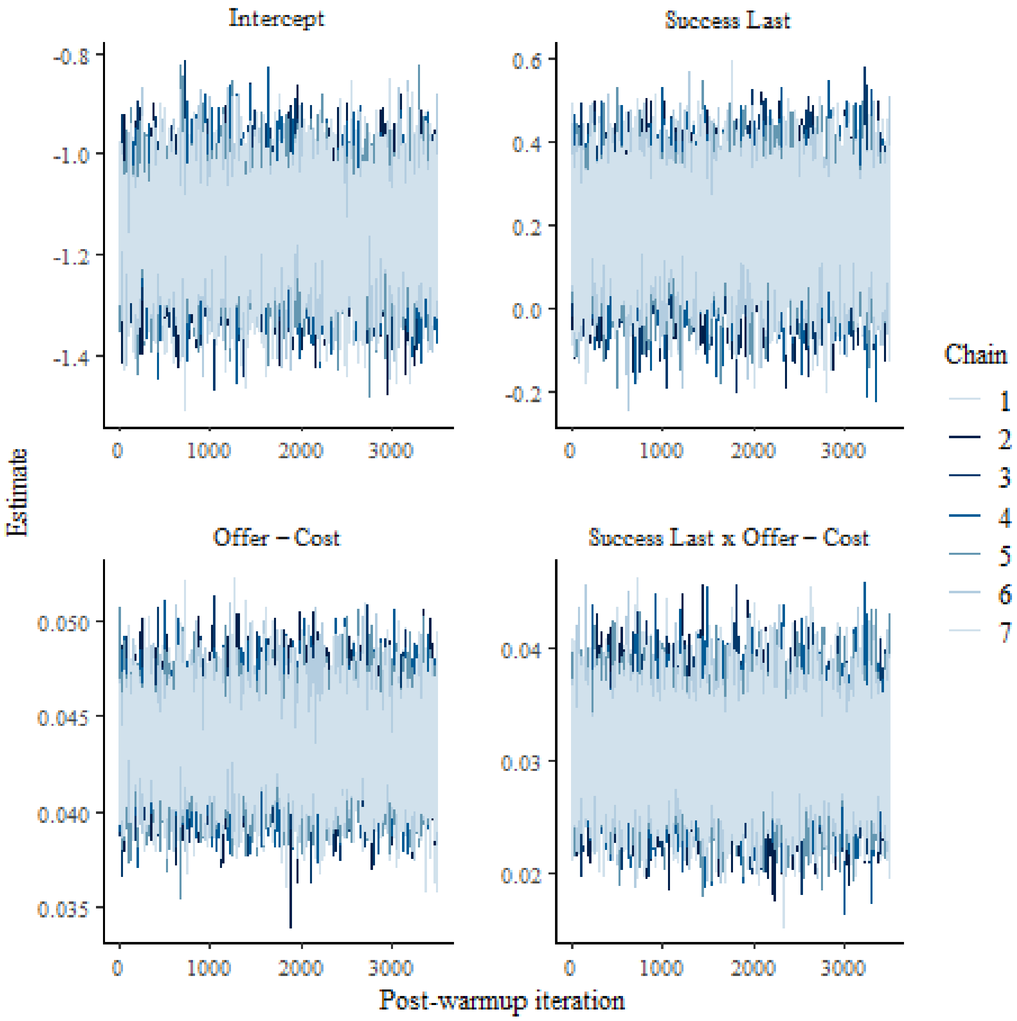

Figure S2. Trace plots for the population-level effects in model 1 fitted in the second block of the robber task. *The plot is a diagnostic plot for Markov chain Monte Carlo (MCMC) estimation showing each draw for the iterations of each chain. The model converged with Rhat = 1 for all estimates except for correlation between the group-level subject's effects of success and offer – cost, here Rhat = 1.02.*

Table S5. Factory task block 1, model 1: population-level effects

	Estimate	Est.Error	l-95% CI	u-95% CI	BF_{10}
Intercept	-1.020	0.078	-1.176	-0.869	-
Success Last	0.195	0.082	0.031	0.354	1.314
Offer - Cost	0.038	0.002	0.035	0.042	5.388e+22
Success x Offer-Cost	0.006	0.002	0.002	0.011	0.101

Table S6. Factory task block 1, model 1: group-level effects

Group		Estimate	Est.Error	l-95% CI	u-95% CI
Subject SD	Intercept	1.088	0.066	0.964	1.224
	Success Last	0.430	0.105	0.223	0.636
	Offer - Cost	0.026	0.002	0.023	0.030
	Success x Offer-Cost	0.018	0.002	0.014	0.022
Subject Corr	Intercept, Success Last	0.548	0.157	0.215	0.825
	Intercept, Offer-Cost	-0.729	0.040	-0.801	-0.647
	Success Last, Offer-Cost	-0.395	0.162	-0.692	-0.061
	Intercept, Success Last x Offer-Cost	0.180	0.107	-0.036	0.382
	Success Last, Success Last x Offer-Cost	-0.199	0.190	-0.536	0.211
	Offer-Cost, Success Last x Offer-Cost	-0.204	0.116	-0.422	0.027
Stimulus SD	Intercept	0.045	0.032	0.002	0.120
	Success Last	0.081	0.059	0.003	0.218
	Offer - Cost	0.001	0.001	0.000	0.002
	Success x Offer-Cost	0.001	0.001	0.000	0.004
Stimulus Corr	Intercept, Success Last	-0.031	0.331	-0.652	0.603
	Intercept, Offer-Cost	-0.086	0.335	-0.688	0.583
	Success Last, Offer-Cost	-0.062	0.331	-0.673	0.579
	Intercept, Success Last x Offer-Cost	0.004	0.331	-0.624	0.632
	Success Last, Success Last x Offer-Cost	-0.047	0.333	-0.661	0.596
	Offer-Cost, Success Last x Offer-Cost	-0.061	0.329	-0.664	0.577

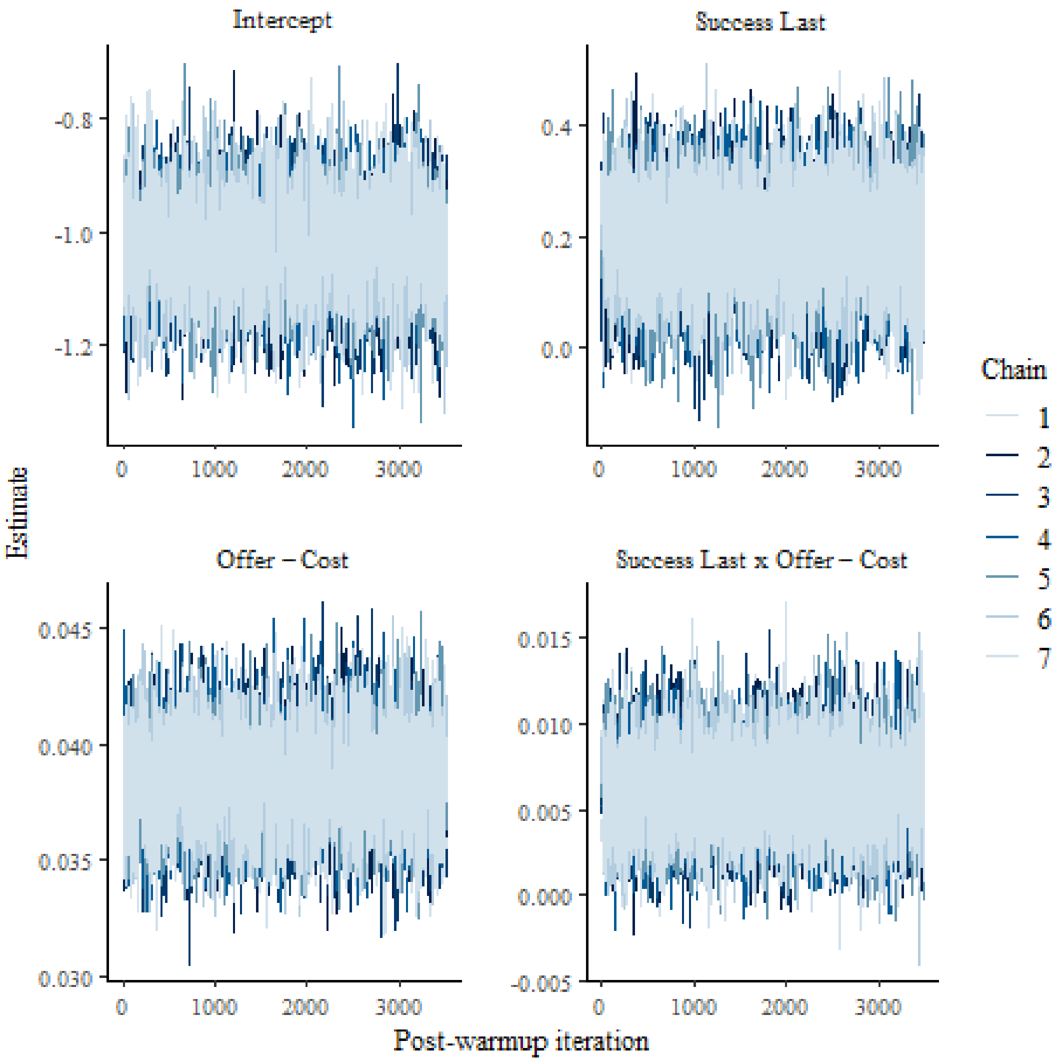

Figure S3. Trace plots for the population-level effects in model 1 fitted in the first block of the factory task. *The plot is a diagnostic plot for Markov chain Monte Carlo (MCMC) estimation showing each draw for the iterations of each chain. The model converged with Rhat = 1 for all estimates except for the correlations between group-level subject's effects of success last and offer-cost, and the correlations between group-level subject's effects of success last and the interaction effect, here Rhat = 1.01.*

Table S7. Factory task block 2, model 1: population-level effects

	Estimate	Est.Error	l-95% CI	u-95% CI	BF_{10}
Intercept	-1.131	0.077	-1.284	-0.984	-
Success Last	0.221	0.094	0.033	0.403	1.377
Offer - Cost	0.038	0.002	0.034	0.041	1.202e+14
Success x Offer-Cost	0.015	0.003	0.010	0.020	7.773e+15

Table S8. Factory task block 2, model 1: group-level effects

Group		Estimate	Est.Error	l-95% CI	u-95% CI
Subject SD	Intercept	1.071	0.066	0.947	1.207
	Success Last	0.653	0.107	0.445	0.868
	Offer - Cost	0.026	0.002	0.023	0.029
	Success x Offer-Cost	0.021	0.002	0.016	0.025
Subject Corr	Intercept, Success Last	0.476	0.136	0.199	0.730
	Intercept, Offer-Cost	-0.729	0.040	-0.800	-0.643
	Success Last, Offer-Cost	-0.245	0.136	-0.508	0.026
	Intercept, Success Last x Offer-Cost	-0.019	0.108	-0.233	0.189
	Success Last, Success Last x Offer-Cost	-0.474	0.127	-0.693	-0.199
	Offer-Cost, Success Last x Offer-Cost	-0.062	0.117	-0.285	0.175
Stimulus SD	Intercept	0.049	0.034	0.002	0.127
	Success Last	0.120	0.076	0.006	0.282
	Offer - Cost	0.002	0.001	0.001	0.003
	Success x Offer-Cost	0.002	0.001	0.000	0.004
Stimulus Corr	Intercept, Success Last	-0.029	0.334	-0.648	0.616
	Intercept, Offer-Cost	-0.293	0.330	-0.810	0.428
	Success Last, Offer-Cost	-0.116	0.312	-0.676	0.522
	Intercept, Success Last x Offer-Cost	-0.053	0.330	-0.661	0.593
	Success Last, Success Last x Offer-Cost	-0.035	0.328	-0.647	0.599
	Offer-Cost, Success Last x Offer-Cost	0.098	0.315	-0.537	0.669

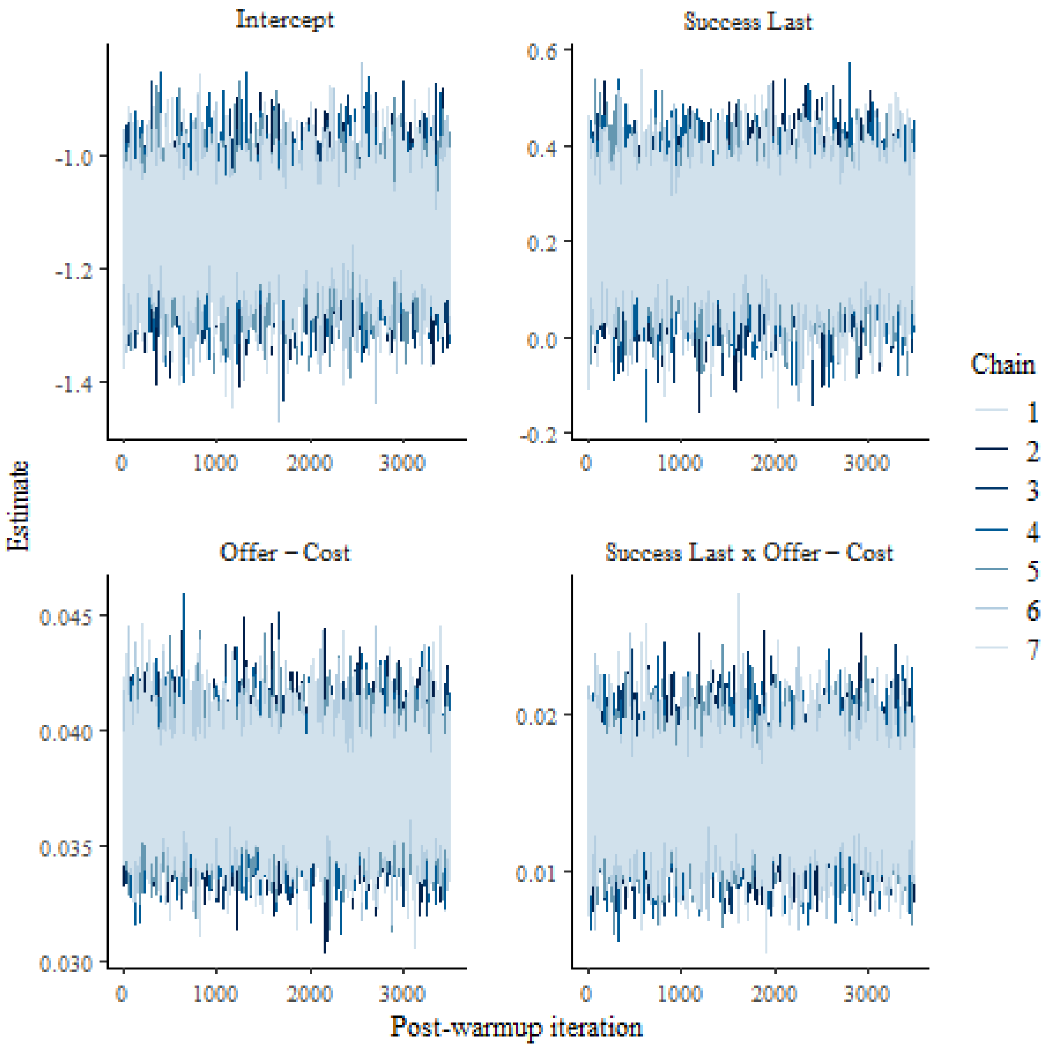

Figure S4. Trace plots for the population-level effects in model 1 fitted in the second block of the factory task. *The plot is a diagnostic plot for Markov chain Monte Carlo (MCMC) estimation showing each draw for the iterations of each chain. The model converged with Rhat = 1 for all estimates.*

Regression model 2, probability of success over trials. Below you find the full results from regression model 2. The model was fitted as a hierarchical Bayesian logistic regression specified as follows:

Choice ~ Intercept + trial number with same stimuli * Success probability of the stimuli +
(Intercept + trial number with same stimuli * Success probability of the stimuli| subject)

Table S9. Robber task block 1, model 2: population-level effects

	Estimate	Est.Error	l-95% CI	u-95% CI	BF_{10}
Intercept	0.7493	0.0811	0.5917	0.9098	-
Trial	-0.1064	0.0231	-0.1517	-0.0614	6.012e+15
Success Prob	0.1940	0.1395	-0.0808	0.4664	0.367
Trial x Success Prob	0.1380	0.0406	0.0589	0.2176	18.737

Table S10. Robber task block 1, model 2: group-level effects

Group		Estimate	Est.Error	l-95% CI	u-95% CI
Subject SD	Intercept	0.466	0.079	0.305	0.614
	Trial	0.138	0.030	0.076	0.192
	Success Prob	0.372	0.190	0.027	0.728
	Trial x Success Prob	0.172	0.056	0.037	0.264
Subject Corr	Intercept, Trial	-0.269	0.209	-0.586	0.229
	Intercept, Success Prob	0.193	0.282	-0.381	0.701
	Trial, Success Prob	-0.140	0.298	-0.673	0.474
	Intercept, Trial x Success Prob	0.190	0.252	-0.391	0.590
	Trial, Trial x Success Prob	-0.729	0.202	-0.928	-0.126
	Trial, Trial x Success Prob	0.023	0.303	-0.539	0.620

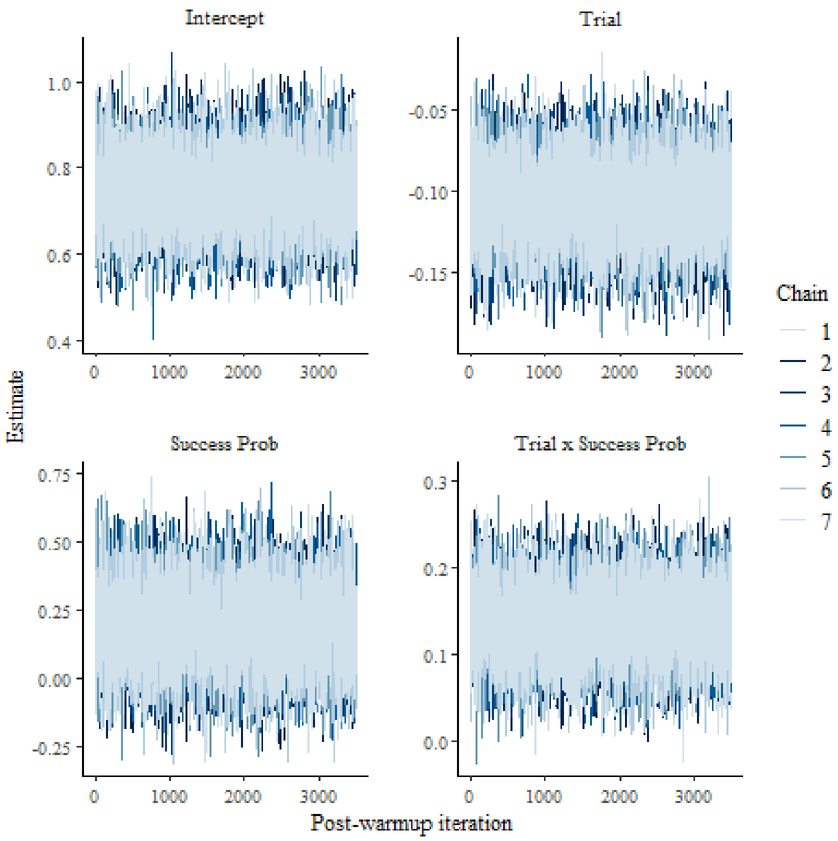

Figure S5. Trace plots for the population-level effects in model 2 fitted in the first block of the robber task. *The plot is a diagnostic plot for Markov chain Monte Carlo (MCMC) estimation showing each draw for the iterations of each chain. The model converged with Rhat = 1 for all estimates.*

Table S11, Robber Task block 2, model 2: population-level effects

	Estimate	Est.Error	l-95% CI	u-95% CI	BF_{10}
Intercept	0.8912	0.0842	0.7267	1.0562	-
Trial	-0.1601	0.0243	-0.2081	-0.1119	4.457e+15
Success Prob	0.0544	0.1393	-0.2162	0.3264	0.146
Trial x Success Prob	0.2111	0.0411	0.1304	0.2921	6.992e+17

Table S12, Robber Task block 2, model 2: group-level effects

Group		Estimate	Est.Error	l-95% CI	u-95% CI
Subject SD	Intercept	0.563	0.070	0.420	0.696
	Trial	0.177	0.028	0.119	0.229
	Success Prob	0.271	0.178	0.012	0.653
	Trial x Success Prob	0.187	0.048	0.070	0.266
Subject Corr	Intercept, Trial	-0.160	0.175	-0.442	0.248
	Intercept, Success Prob	0.059	0.302	-0.526	0.633
	Trial, Success Prob	-0.183	0.317	-0.725	0.480
	Intercept, Trial x Success Prob	0.217	0.205	-0.256	0.556
	Trial, Trial x Success Prob	-0.746	0.152	-0.913	-0.348
	Trial, Trial x Success Prob	0.055	0.315	-0.548	0.652

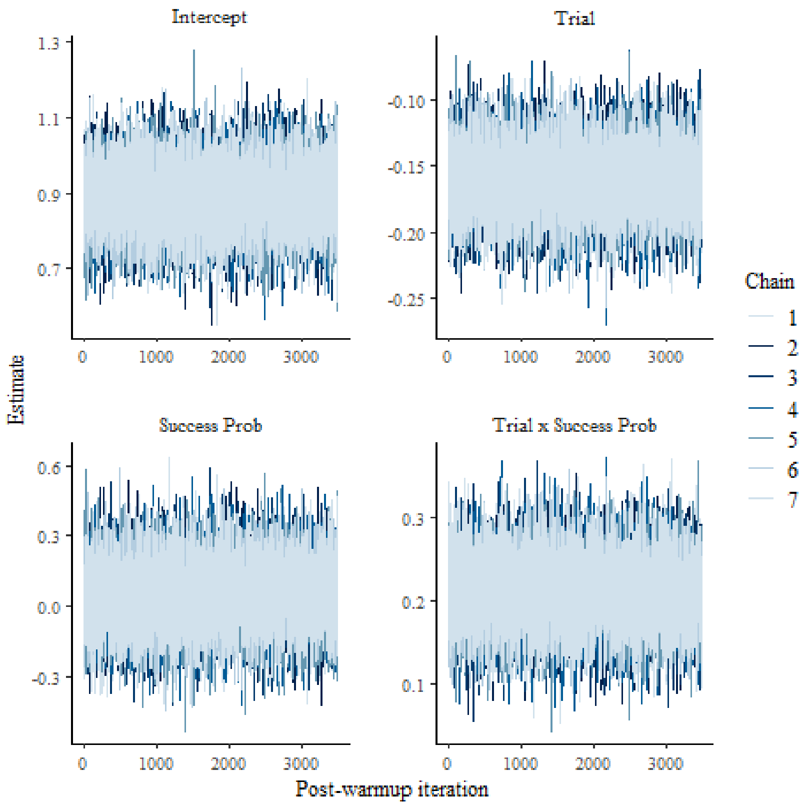

Figure S6. Trace plots for the population-level effects in model 2 fitted in the second block of the robber task. *The plot is a diagnostic plot for Markov chain Monte Carlo (MCMC) estimation showing each draw for the iterations of each chain. The model converged with Rhat = 1 for all estimates, except for group-level effect for success probability, here Rhat = 1.01.*

Table S13. Factory Task block 1, model 2: population-level effects

	Estimate	Est.Error	l-95% CI	u-95% CI	BF_{10}
Intercept	0.5925	0.0775	0.4410	0.7444	-
Trial	-0.0651	0.0205	-0.1051	-0.0247	3.299
Success Prob	-0.0340	0.1309	-0.2909	0.2223	0.135
Trial x Success Prob	0.0776	0.0360	0.0072	0.1476	0.337

Table S14. Factory Task block 1, model 2: group-level effects

Group		Estimate	Est.Error	l-95% CI	u-95% CI
Subject SD	Intercept	0.497	0.072	0.353	0.640
	Trial	0.099	0.027	0.050	0.155
	Success Prob	0.445	0.161	0.073	0.718
	Trial x Success Prob	0.077	0.050	0.004	0.183
Subject Corr	Intercept, Trial	-0.522	0.167	-0.765	-0.129
	Intercept, Success Prob	0.083	0.255	-0.376	0.603
	Trial, Success Prob	-0.351	0.256	-0.772	0.233
	Intercept, Trial x Success Prob	0.025	0.308	-0.594	0.575
	Trial, Trial x Success Prob	-0.368	0.351	-0.856	0.425
	Trial, Trial x Success Prob	0.059	0.318	-0.557	0.657

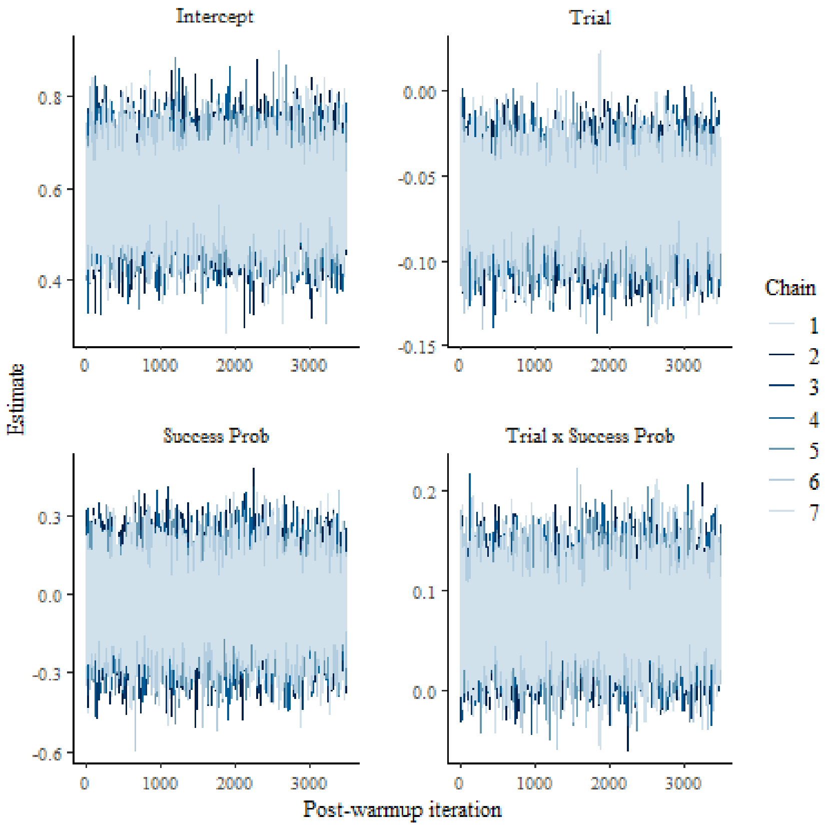

Figure S7. Trace plots for the population-level effects in model 2 fitted in the first block of the factory task. *The plot is a diagnostic plot for Markov chain Monte Carlo (MCMC) estimation showing each draw for the iterations of each chain. The model converged with Rhat = 1 for all estimates except for the group-level effects for trial, success probability and the interaction effect, here Rhat = 1.01.*

Table S15. Factory task block 2, model 2: population-level effects

	Estimate	Est.Error	l-95% CI	u-95% CI	BF_{10}
Intercept	0.5022	0.0751	0.3557	0.6515	-
Trial	-0.0824	0.0201	-0.1219	-0.0432	151.968
Success Prob	0.0523	0.1305	-0.2002	0.3082	0.148
Trial x Success Prob	0.1170	0.0359	0.0467	0.1863	12.242

Table S16. Factory task block 2, model 2: group-level effects

Group		Estimate	Est.Error	l-95% CI	u-95% CI
Subject SD	Intercept	0.419	0.064	0.292	0.545
	Trial	0.070	0.027	0.013	0.124
	Success Prob	0.493	0.138	0.184	0.737
	Trial x Success Prob	0.067	0.046	0.003	0.169
Subject Corr	Intercept, Trial	-0.149	0.249	-0.566	0.400
	Intercept, Success Prob	0.006	0.256	-0.439	0.547
	Trial, Success Prob	-0.423	0.251	-0.811	0.154
	Intercept, Trial x Success Prob	-0.064	0.305	-0.646	0.529
	Trial, Trial x Success Prob	-0.296	0.362	-0.853	0.476
	Trial, Trial x Success Prob	-0.047	0.326	-0.655	0.593

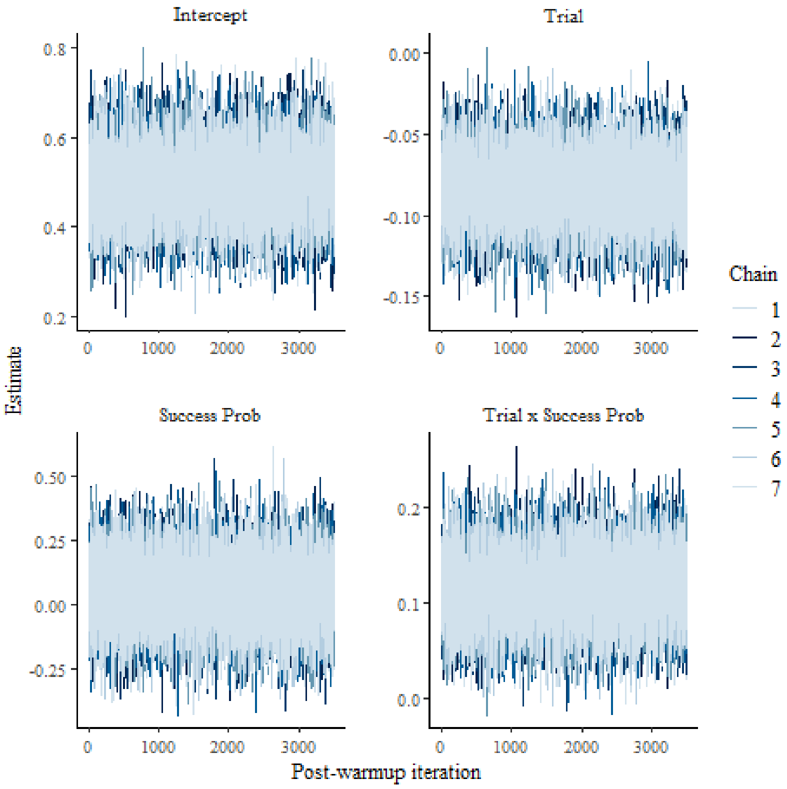

Figure S8. Trace plots for the population-level effects in model 2 fitted in the second block of the factory task. *The plot is a diagnostic plot for Markov chain Monte Carlo (MCMC) estimation showing each draw for the iterations of each chain. The model converged with Rhat = 1 for all estimates except for the group-level effect of trial and the interaction effect, here Rhat = 1.01.*

Modeling analysis

The fit of the factory task is improved by a lapse parameter. We next explored augmentation of the winning models in each block and task to explain away potential nuisance effects and improve the estimation of the prior. As stated in our preregistration, we made stepwise model comparisons between the winning basic model and augmented versions of that model. For these analyses we did not have a preregistered hypothesis.

We first considered two augmentations. One applied to the choice function for both models: adding a bias parameter allowing for a lapse in attention. This was done by introducing a probability that the choice is not based on the value difference between the active and the passive choices. The support for this parameter was -1 to 1, whereby values of < 0 of this φ parameter implements a passivity bias and $\varphi > 0$ implements an action bias (see Methods).

The second augmentation only applied to the Bayesian model: removing the resetting of the subjective probability in the Bayesian learner model for new stimuli, thus causing the model to update the subjective probability continuously throughout the task, independent of the stimulus.

The result from the stepwise model comparisons established that the best fitting Bayesian model resets to the prior each time a new stimulus is encountered, thereby rejecting the inclusion of continuous learning (Robber, block 1: *PXP* = 0.272; block 2 *PXP* = 0.017; Factory, block 2: *PXP* = 0.008). Furthermore, the fit of the Bayesian learner was also not improved by including a lapse parameter in the robber task (Block 1: *PXP* = 0.336; Block 2: *PXP* = 0). In contrast, the fit of the models in the factory task was improved by including this bias parameter in both the first block with the Static probability model (*PXP* = 0.997) and in the second block with the Bayesian learner (*PXP* = 0.543; see Table S17). As the value of the lapse parameter is positive on average, participants tend to be more biased towards the active choice in both the first (*M* = 0.104, *SD* = 0.159) and second block (*M* = 0.069, *SD* = 0.162) of the factory task.

These results show that the choice data in the robber task was best fitted by the basic Bayesian learner model whereas the choice data in the factory task was best fitted by a model that included an action bias on top of the Static probability model in block 1 and the basic Bayesian model in block 2. This is a result that was not observed in our previous pilot (see Supplementary Note 2, pilot data).

Table S17. Model comparison for augmented models.

Block	Task		Bayes vs. Continuous Updating		Bayes vs. Bidirectional Bias		Static Probability vs. Bidirectional Bias	
Block 1	Robber	*M.freq*	**0.5182**	0.4818	**0.5127**	0.4873		
		PXP	**0.7279**	0.2721	**0.6642**	0.3358		
	Factory	*M.freq*					0.4163	**0.5837**
		PXP					0.0026	**0.9974**
Block 2	Robber	*M.freq*	**0.5632**	0.4368	**0.6201**	0.3799		
		PXP	**0.9825**	0.0175	**1.0000**	0.0000		
	Factory	*M.freq*	**0.5725**	0.4275	0.4968	**0.5032**		
		PXP	**0.9923**	0.0077	0.4569	**0.5431**		

Task-invariance. Below the full results from model comparison between 1 and 2 prior models is shown.

Table S18. Model comparison between 1- and 2 - prior Bayesian models.

Session		1-Prior + Factory Bidirectional Bias	2-Prior + Factory Bidirectional Bias
Block 1	*M.freq*	**0.5772**	0.4228
	PXP	**0.9951**	0.0049
Block 2	*M.freq*	**0.6901**	0.3099
	PXP	**1.0000**	0.0000
Follow-Up	*M.freq*	**0.5724**	0.4276
	PXP	**0.9263**	0.0737

Alternative reliability measures. Reliability was measured in three different ways in the study, explained variance (R^2), Spearman's correlation (r_s) and with two-way mixed effects, absolute agreement, single measure, inter-class correlations (ICC(2,1)). All measures were carried out in R. To calculate the ICC the R package *irr* v. 0.84.1[1] was used. The explained variance was calculated as follows:

(1) $$R^2 = 1 - \frac{MSE}{VAR_y}$$

Where *VAR* is the variance and *MSE* is mean squared error and is defined as:

(2) $$MSE = \frac{1}{N}\sum (y_i - x_i)^2$$

Where y_i is the subject's parameter value at time 1 and x_i is the parameter at time 2. Results are presented below:

Table S19. Test retest, block 1 and 2.

Parameters	R^2	r_s	ICC(2,1)
μ_0	0.410	0.678	0.691
σ_0^2	0.313	0.554	0.591
T^R	0.090	0.605	0.630
T^F	-0.209	0.586	0.619
φ	-0.383	0.374	0.487

Table S20. Test retest, block 1 and follow-up.

Parameters	R^2	r_s	ICC(2,1)
μ_0	0.542	0.761	0.748
σ_0^2	0.130	0.627	0.645
T^R	0.145	0.624	0.583
T^F	-0.246	0.492	0.470
φ	0.110	0.214	0.387

Table S21. Test retest, block 2 and follow-up.

Parameters	R^2	r_s	ICC(2,1)
μ_0	0.542	0.760	0.746
σ_0^2	0.130	0.636	0.652
T^R	0.145	0.624	0.584
T^F	-0.246	0.492	0.469
φ	0.110	0.204	0.390

Robustness Checks and Sensitivity Analyses

The parameter and model recovery were performed with 1000 simulations with parameters sampled from the mean and standard deviation of the fitted parameters on the data. Parameter recovery was performed on the winning models with 1000 simulations, utilizing HBI[2]. Model recovery was performed on all candidate models. For model recovery, we constructed confusion matrixes with the models that were pitted against each other in model comparisons using HBI[1] to make sure that the models were not confused. If an augmented model was confused with a basic model, the augmented model was dropped from model comparisons. The model was considered robust if the model frequency of the generative model in the model recovery was above 0.65 in a two-model comparison and above 0.6 in a four-model comparison if no other model had a model frequency above 0.3. The parameter and model recovery were performed for both tasks separately and together. Below are the full results from the model and parameter recovery.

Model recovery. Model recovery was carried out by simulating data with each of the target models with parameters sampled from the observed group-level mean and standard error for each of the models when fitted on the data. For confusion matrices where more than two models were present, we sampled the parameters from the winning model from the full model comparison. For the other models, parameters were sampled after fitting that specific model to all participants using HBI. For confusion matrices where only two models were present, parameters were sampled after fitting that specific model to all participants using HBI regardless of which was the winning model. Thereby giving the best estimation of the potential parameters that the participants would exhibit under each model. The estimated group-level mean and standard deviation were used to create a Gaussian distribution of each parameter from which 1000 samples were randomly drawn and data was simulated. A model comparison between the candidate models was then performed with HBI. The results of each model comparison are presented in Figures S9 to S13. Results show that overall, the predictions of each model are distinct and simulations from each model are not confused with other models within each model comparison. However, we observed that the Bayesian learner model is slightly confused with the Static probability when we sample from the observed parameters in block 1 (Fig. S9b) but not when sampling from the parameters observed in block 2 (Fig. S10b). We also observed that the continuous learning Bayesian model is slightly confused with the basic Bayesian model as in all analysis the observed frequency of the continuous learning model is just under our predetermined threshold of 0.65 (Fig. S11a and S12).

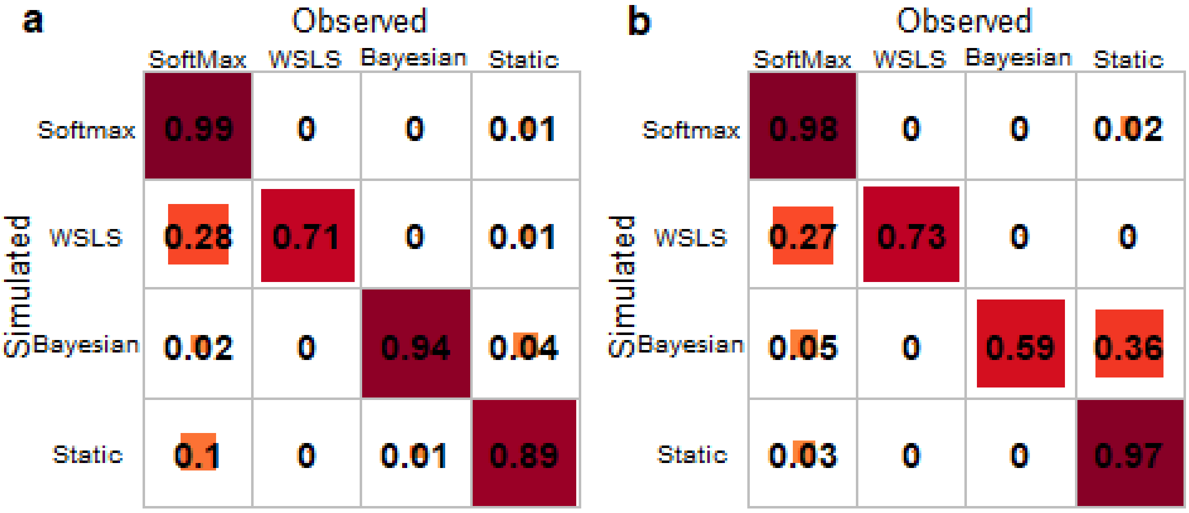

Figure S9. Model recovery of basic models in block 1. *Figure shows the confusion matrix for the basic models fitted to each task separately, with parameters extracted from data in the first block of the tasks. Numbers reported are the model frequency from 1000 simulations.* ***a)*** *Shows the models in the robber task.* ***b)*** *Shows the models in the factory task.*

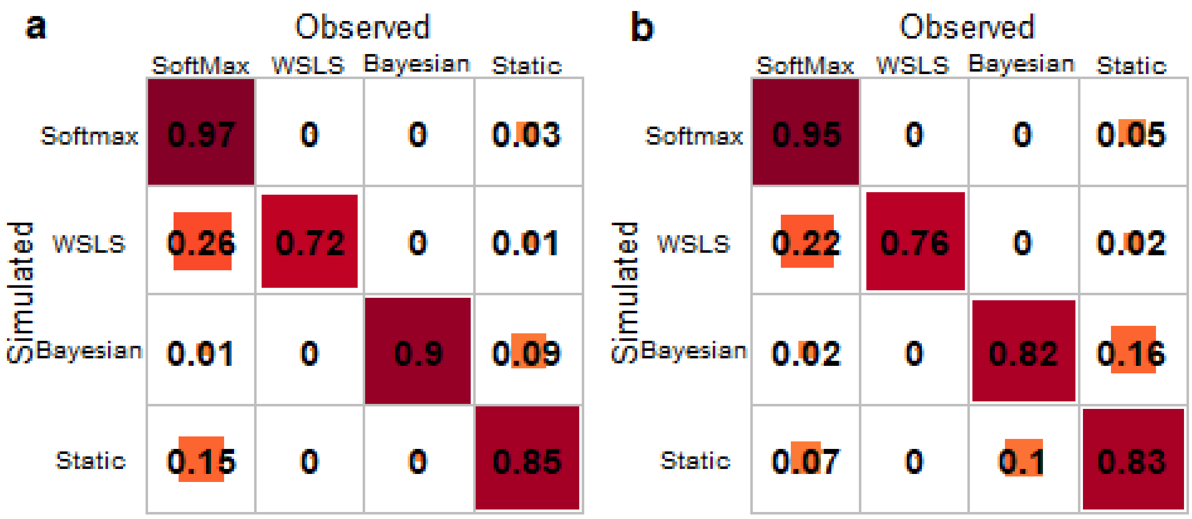

Figure S10. Model recovery of basic models in block 2. *Figure shows the confusion matrix for the basic models fitted to each task separately, with parameters extracted from data in the second block of the tasks. Numbers reported are the model frequency from 1000 simulations.* ***a)*** *Shows the models robber task.* ***b)*** *Shows the models in the factory task.*

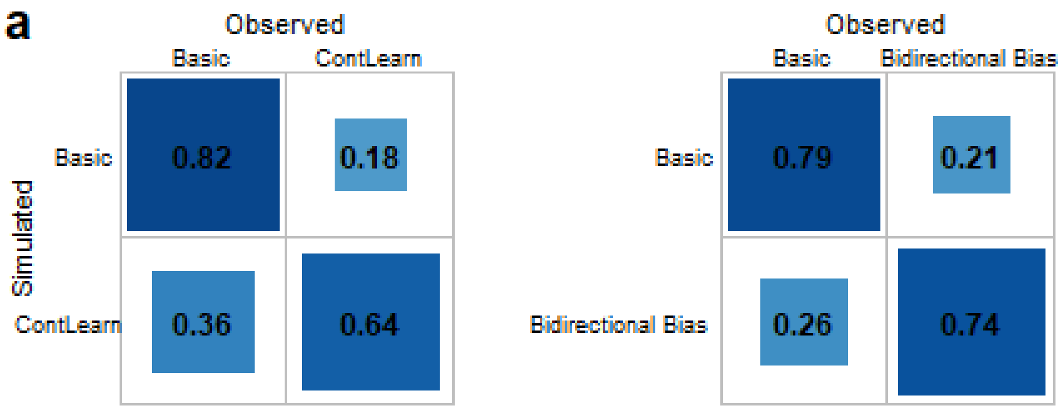

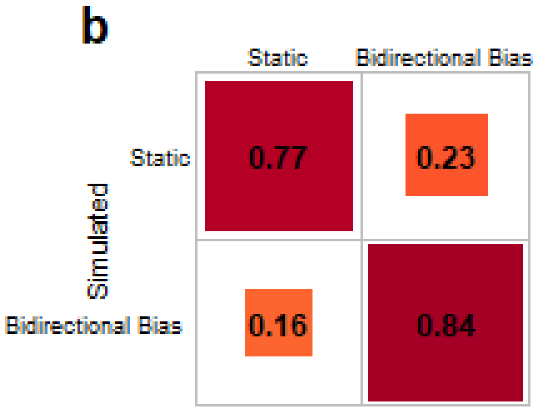

Figure S11. Model recovery of augmented models in block 1. *Figure shows the confusion matrix for the augmented models fitted to each task separately. Numbers reported are the model frequency from 1000 simulations.* ***a)*** *Shows the models in the robber task.* ***b)*** *Shows the models in the factory task.*

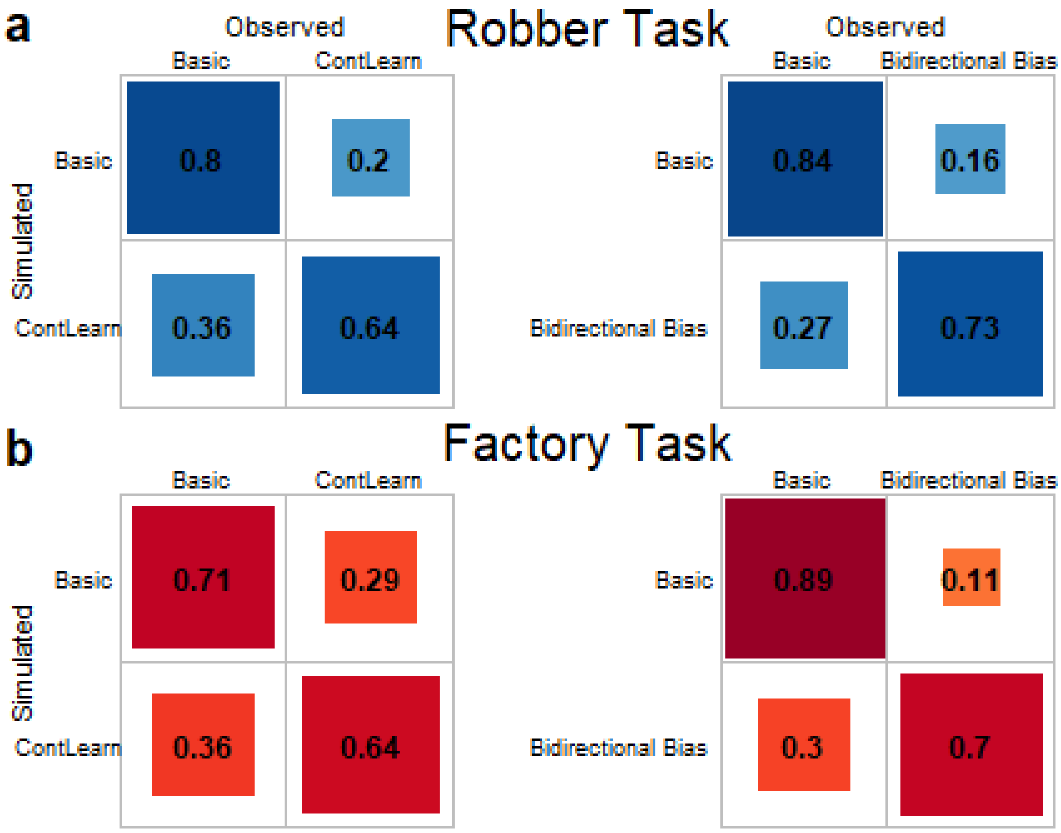

Figure S12. Model recovery of augmented models in block 2. *Figure shows the confusion matrix for the augmented models fitted to each task separately. Numbers reported are the model frequency from 1000 simulations.* ***a)*** *Figure shows the models robber task.* ***b)*** *Figure shows the models in the factory task.*

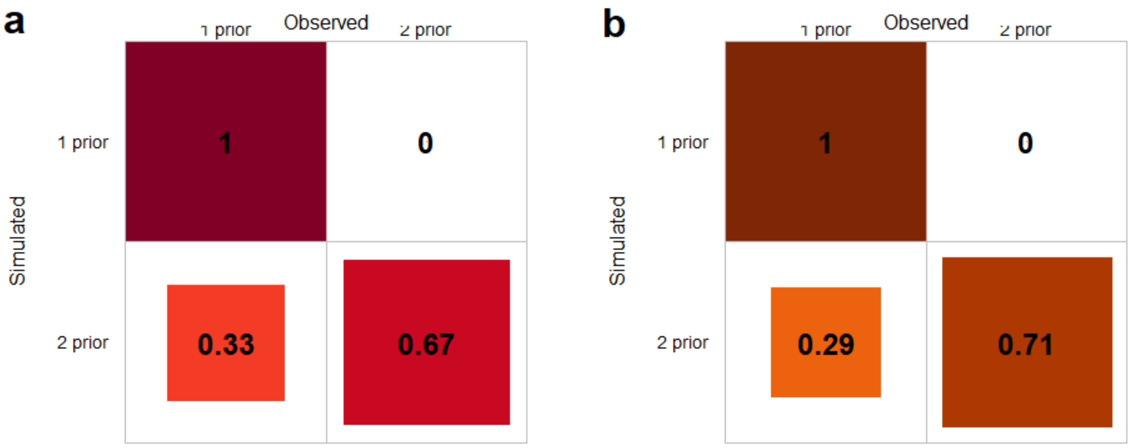

Figure S13. Model recovery of models fitted to both tasks. *Figure shows the confusion matrix for the 1 and 2 prior models fitted to each block separately. Numbers reported are the model frequency from 1000 simulations.* ***a)*** *Figure shows the models fitted with parameters from the first block.* ***b)*** *Figure shows the models fitted with parameters from the second block.*

Parameter recovery. Parameter recovery was only carried out for the winning models within each task and for the tasks modeled together. This was for block 1 the basic Bayesian model in the robber task, the Static probability model with action bias in the factory task and the one task-invariant prior model with the two *invT* parameters and action bias in the factory task.

Parameter recovery was carried out by sampling the group-level mean and standard error for each of the parameters of the models from the data. The means and standard error were taken from forcing the target model to be fitted to all participants in HBI. We then draw 1000 random samples from a normal distribution of the parameter values constructed with the group level mean and the group level standard deviation transformed from the standard error so: $SD = SEM$ * sqrt(N_{total}). Choice data was then simulated with these parameters and fitted using the target model. A Spearman's correlation (r_s) and explained variance (R^2) were then calculated between the parameter values used in the simulation and the recovered parameter values when only the target model was fitted using HBI.

Parameter recovery showed strong correlations between simulated and recovered parameters. This indicates that for our winning models, we are able to recover the participants' priors (see Fig. S14, S15 and Table S22 and S23 for full results).

Table S22. Parameter recovery block 1

Parameter	Task	R^2	r_s
μ_0	Robber	0.6975	0.8562
p	Factory	0.7479	0.8809
μ_0	Both Tasks	0.8538	0.9154
σ_0^2	Robber	0.616	0.8309
σ_0^2	Both Tasks	0.7595	0.7961
T	Robber	0.4945	0.92
T	Factory	-4.4012	0.8628
T^R	Both Tasks	0.3009	0.9072
T^F	Both Tasks	-1.6609	0.901
φ	Factory	0.3266	0.8044
φ	Both Tasks	0.8156	0.901

Table S23. Parameter recovery block 2

Parameter	Task	R^2	r_s
μ_0	Robber	0.8053	0.8853
μ_0	Factory	0.5834	0.8186
μ_0	Both Tasks	0.8756	0.9209
σ_0^2	Robber	0.5278	0.7786
σ_0^2	Factory	0.3327	0.6887
σ_0^2	Both Tasks	0.7567	0.8345
T	Robber	0.2534	0.9055
T	Factory	-2.9924	0.817
T^R	Both Tasks	0.3195	0.9159
T^F	Both Tasks	-1.6609	0.901
φ	Factory	0.3348	0.8132
φ	Both Tasks	0.7702	0.8806

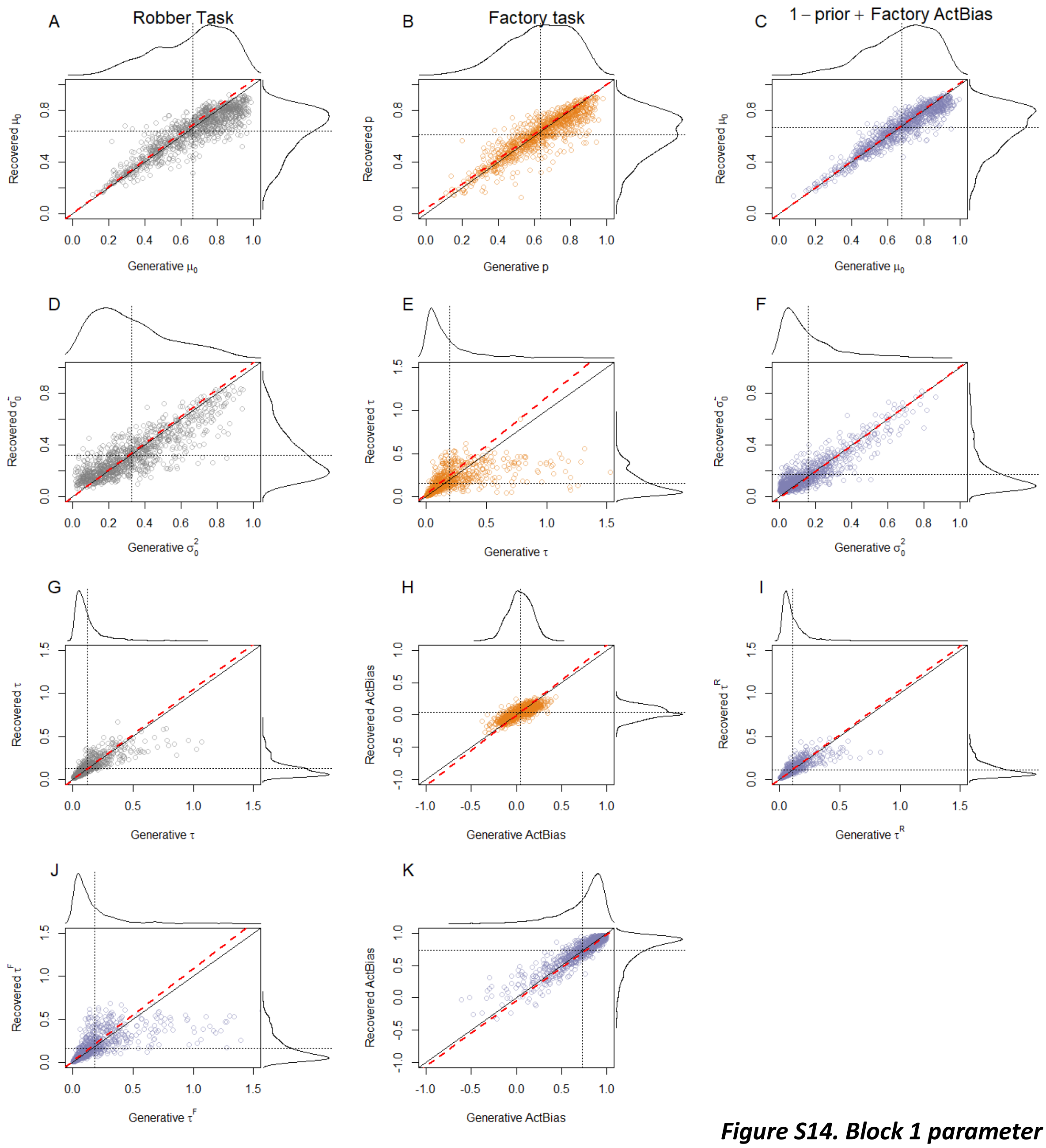

Figure S14. Block 1 parameter recovery. *Each plot shows the simulated and observed parameters for each of the winning models in the first block.* Each dot corresponds to a simulated data set, indicating the generative and the recovered value for a given parameter, *the dotted lines show the mean of the simulated and observed parameter, and the diagonal indicates a perfect correlation. The red dashed lines indicate the regression line.* ***a****) Correlation between the simulated and observed μ_0 parameter in the basic Bayesian learner model in block 1 of the robber task.* ***b****) Correlation between the simulated and observed p parameter in the Static probability model with action bias in block 1 of the factory task.* ***c****) Correlation between the simulated and observed μ_0 parameter in the task-invariant 1 prior Bayesian learner model with a bias parameter in the factory task in block 1 of both tasks.* ***d****) Same as a but for the σ_0^2 parameter.* ***e****) Same as b but for τ the parameter.* ***f****) Same as c but for the σ_0^2 parameter.* ***g****) Same as a but for the τ parameter.* ***h****) Same as b but for the bias (φ) parameter.* ***i****) Same as c but for the τ parameter in the robber task.* ***j****) Same as c but for the τ parameter in the factory task.* ***k****) Same as c but for the bias (φ) parameter in the factory task.*

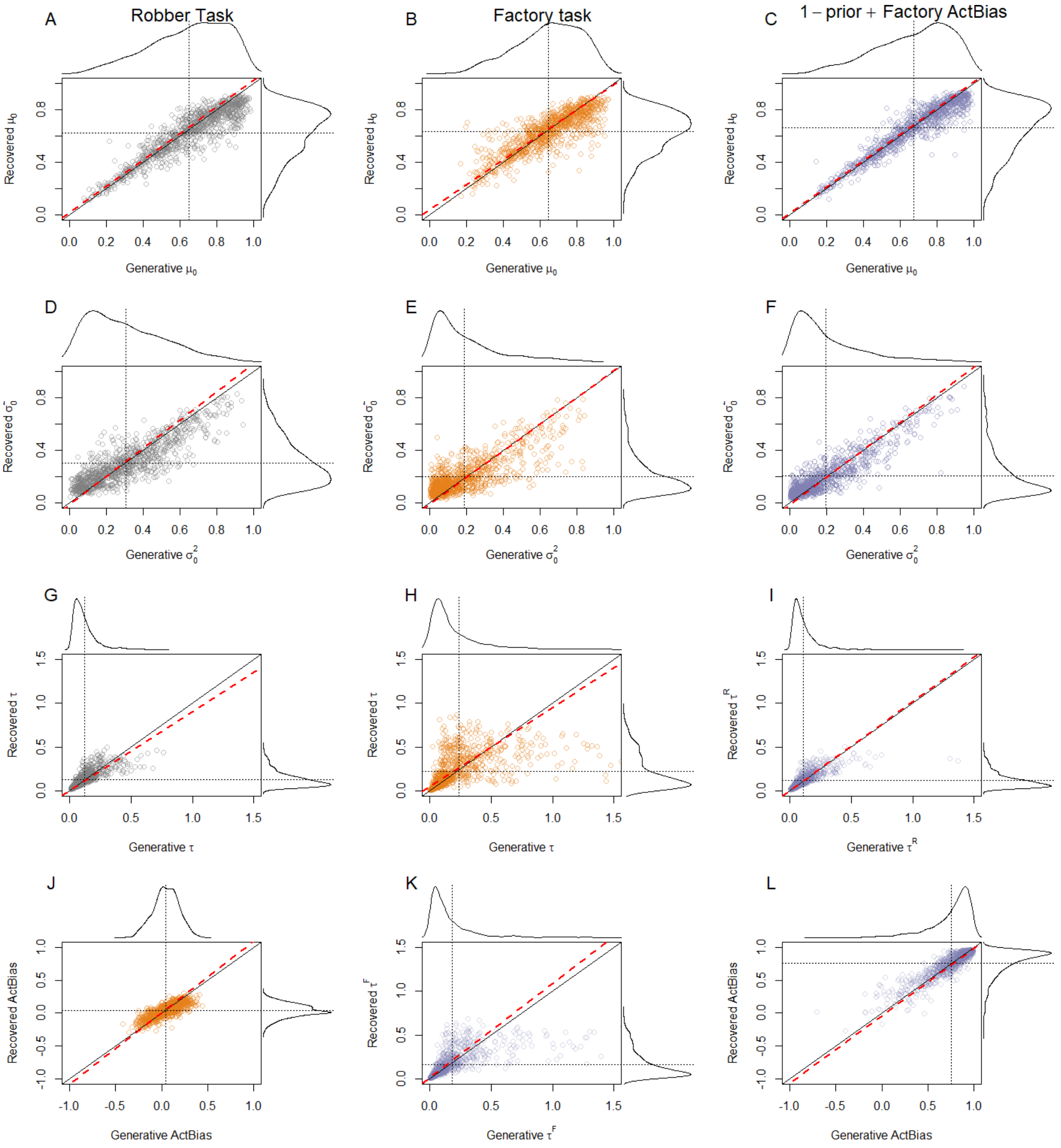

Figure S15. Block 2 parameter recovery. *Each plot shows the simulated and observed parameters for each of the winning models in the second block.* Each dot corresponds to a simulated data set, indicating the generative and the recovered value for a given parameter, *the dotted lines show the mean of the simulated and observed parameter, and the diagonal indicates a perfect correlation. The red dashed lines indicate the regression line.* ***a****) Correlation between the simulated and observed μ_0 parameter in the basic Bayesian learner model in block 2 of the robber task.* ***b****) Correlation between the simulated and observed μ_0 parameter in the Bayesian learner model with a bias parameter in block 2 of the factory task.* ***c)*** *Correlation between the simulated and observed μ_0 parameter in the task-invariant 1 prior Bayesian learner model with a bias parameter in the factory task in block 2 of both tasks.* ***d)*** *Same as a but for the σ_0^2 parameter.* ***e)*** *Same as b but for the σ_0^2 parameter.* ***f)*** *Same as c but for the σ_0^2 parameter.* ***g)*** *Same as a but for the τ parameter.* ***h)*** *Same as b but for the τ parameter.* ***i)*** *Same as c but for the τ parameter in the robber task.* ***j)*** *Same as B but for the action bias (φ) parameter.* ***k)*** *Same as c but for the τ parameter in the factory task.* ***l****) Same as c but for the bias (φ) parameter in the factory task.*

Supplementary Methods

Participants

Below is a full breakdown of the sample characteristics of the 279 participants who participated in the current study. The median completion time of the participants in the first session was 1 hours and 34 minutes, the median completion time for the follow-up session was 1 hour. To participate, participants had to be above the age of 18 and below the age of 40. Complete self-reported demographic data were collected for all but 4 participants. The final sample for the first day had a mean age of 27.12 (*SD* = 5.06) years with 133 females and 142 males and 36 different nationalities reported: Australia (N = 1), Bulgaria (N = 1), Canada (N = 3), Chile (N = 8), China (N = 2), Croatia (N = 1), Czech Republic (N = 2), Estonia (N = 1), Finland (N = 2), France (N = 1), Germany (N = 1), Greece (N = 8), Hungary (N = 10), Israel (N = 3), Italy (N = 11), Latvia (N = 3), Mexico (N = 17), Netherlands (N = 4), New Zealand (N = 1), Nigeria (N = 2), Norway (N = 1), Philippines (N = 1), Poland (N = 38), Portugal (N = 40), Russian Federation (N = 1), Serbia (N = 1), Slovenia (N = 2), South Africa (N = 88), Spain (N = 2), Sweden (N = 3), Turkey (N = 2), Ukraine (N = 1), United Kingdom (N = 11), United States (N = 1), Vietnam (N = 1), Zimbabwe (N = 2). Participants' mean number of previous approved submissions on prolific was 234.88 (SD = 248.86, median = 176, min = 0, max = 2559).

Questionnaires

Participants of the study were asked to fill out 12 questionnaires. As stated in the preregistration, only the positive affect subscale of the PANAS[3] and the trait subscale of the STAI Y-form 2[4] were of interest for the current study. The rest of the questionnaires will be used in a follow-up study. Participants were asked to fill out the following questionnaires.

Depression. Participants filled out 4 different questionnaires measuring symptoms of depression. These were chosen based on low item overlap and high specificity to cover the full spectrum of depressive symptoms. The questionnaires are the following: The Patient Health Questionnaire-9 (PHQ9)[5], the Center for Epidemiologic Studies Depression Scale (CES-D)[6], the Inventory for Depressive Symptomatology, Self-Report (IDS-SR)[7], and the Zung Self-Rating Depression Scale (SDS)[8].

Anxiety. Participants filled out 3 questionnaires measuring anxiety and worry. The questionnaires are the following: The State-Trait Anxiety Questionnaire (STAI Y-form 2)[4], the Penn State Worry Questionnaire (PSWQ)[9], and the GAD-7[10] anxiety scale.

Anhedonia/Motivation. Participants filled out 3 questionnaires measuring apathy and motivation. The questionnaires are the following: The BIS/BAS scales[11], the Apathy Motivation Index[12], and the Snaith-Hamilton Pleasure Scale (SHAPS)[13].

Lastly, participants filled out a questionnaire concerning general affect: The Positive and Negative Affect Schedule (PANAS)[3] and general health: The RAND 36-item health survey 1.0[14].

Implementation of action bias

The text below describes how the implementation of the *bias* parameter in the action bias models. To ensure that we could estimate the likelihood as a function of bias using gradient-based optimization, we replaced the rectified linear (whose gradient is undefined at $\varphi = 0$) with a SoftPlus function with the parameter alpha set at 100. This was implemented as follows:

(3) $$[\varphi]_+ \approx \text{SoftPlus}(\varphi, \text{alpha}) = \frac{1}{\text{alpha}} \log(1 + e^{\text{alpha} \cdot \varphi})$$

further adjusted so that for values from -1 to 1, it returns values running exactly between 0 and 1:

(4) $[\varphi]_+ \approx \text{AdjSoftPlus}(\varphi) = \text{SoftPlus}(\varphi) - \text{SoftPlus}(1) + 1$

Similarly, we noted that the absolute value function (whose gradient is also undefined at $\varphi = 0$) can be rewritten using the rectified linear function, and as such it can also be approximated by AdjustSoftPlus as:

(5) $|\varphi| = [\varphi]_+ + [-\varphi]_+ \approx \text{AdjustSoftPlus}(\varphi) + \text{AdjustSoftPlus}(-\varphi)$

These approximations are then used to reimplement the SoftMax choice function in equation (2) of the main text as follows:

(6) $$P_t(\text{go}) = \text{AdjSoftPlus}(\varphi) + (1 - (\text{AdjSoftPlus}(\varphi) + \text{AdjSoftPlus}(-\varphi))) \cdot \frac{1}{1+e^{-(Q_t(\text{go})-Q_t(\text{ng}))/\tau}}$$

Supplementary Note 2

Pilot Data

Before the main study a pilot study was conducted to test the method and models of the study. The results from this pilot study were largely replicated in the main study. Below we report the method and results from that pilot study.

Method and results

Methods overview

We recruited 73 participants from the online participant pool prolific.co (www.prolific.co) to complete the experiment online on their own computer. The experiment was hosted on pavlovia.org. Out of the 73 participants, 60 reported that they completed the whole experiment. Out of these, the data of 1 participant was missing. 5 participants were also excluded from the analysis because they had failed more than 2 attention checks. As a result, 54 participants were included in the analysis. All participants provided informed consent before starting the experiment and were compensated with 10 £ if they completed the experiment without failing more than 2 attention checks. To incentivise participants, an additional bonus of up to 1 £ was given based on their performance on a randomly selected trial in each task and block resulting in a maximum bonus of 4 £. The bonus was provided after the completion of the whole experiment.

Participants carried out the experiment as stated in the main text with some minor exceptions. In the pilot experiment participants only performed 3 practice trials before beginning the tasks. However, participants performed these practice trials before each block of the task. Furthermore, the written information about the task was repeated before each block of the task. In the main experiment, this information was only provided before beginning the first block of the tasks on both the first day and the follow-up. Beyond this change, the telegraphing of the successes and losses was made more legible after this pilot by making the font size bigger and bold. We also changed the colour of the outcomes in the factory task from red to green when the participant successfully repaired the machine. Thereby distinguishing losses (red) and successes (green) instead of losing and gaining points as was the case in this pilot.

Model agnostic results. We analysed the participants' choice behaviour in both blocks of the tasks with two different cross-classified hierarchical Bayesian logistic regressions in each task and block. Models were fit using the package *brms*[15,16] v.2.19.0 in R v.4.3.0 (2023-04-21 ucrt). The models were fit with weakly regularizing Gaussian prior with $M = 0$ and varying standard deviations for the

intercept (*SD* = 1.5), population (*SD* = 1) and group-level effects (*SD* = 1). The prior for the group-level effects' correlation matrices were a Lewandowski-Kurowicka-Joe (LKJ) distribution with the parameter set at 3, thereby somewhat favouring weaker correlations[17,18]. Models were fit with 4500 iterations in 7 chains with 1000 samples in each chain being discarded as warm-up. This gave a total of 24,500 samples for the posterior estimate. The regression models were run in each task and block of the tasks separately.

Two regression models were fit, mirroring the models in the main text. One model estimates the effects of trial-wise offers - costs and the impact of the last trial being a successful action on the probability of performing an active choice. This model (regression model 1) was fit hierarchically cross-classified with random slopes and intercepts in subjects and stimuli. The estimates of the population-level effect of the first model can be seen in Fig. S16a and Bayes factors (BF_{10}) associated with each estimate is presented in Table S23. The second model estimated the effects of learning within stimuli by estimating the effect of trial within stimuli and the effect of the underlying probability of success when choosing the active action with a specific stimulus. This model was only fitted hierarchically with trials within subjects. The estimates of the population-level effects of this model can be seen in Fig. S16 and Bayes factors associated with each estimate is presented in Table S24.

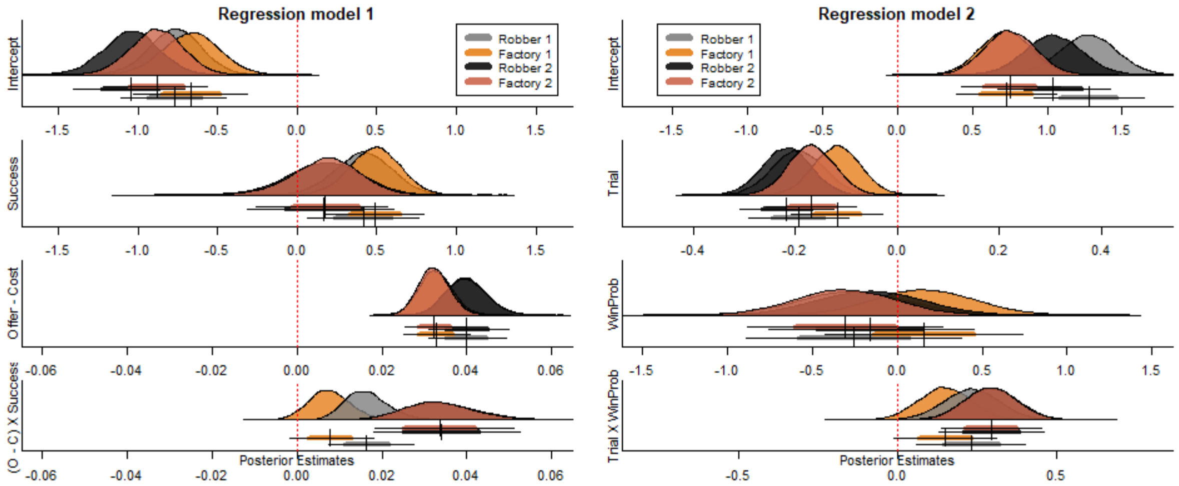

Figure S16. Population-level effects of the regression models. *Plots show the posterior estimates for the population-level effects from the fitted regression models. For each population-level effect, the distribution of the posterior draws is plotted along with the mean estimate as the vertical line. The horizontal coloured line shows the error of estimate and the horizontal black line shows the range of the 95 % credibility interval. The dotted red line indicates a posterior estimate of 0. Note that the scale of the x-axis varies for the estimates, and the y-values of the distributions are arbitrary but held constant within estimated effects but not within models.* ***a****) Shows the population-level posterior estimates of the first logistic regression model (Regression model 1) fitted separately in the different blocks and tasks. Beyond the Intercept the following population-level effects on choice (active = 1, passive = 0) were estimated: the effect of the previous trial being a success (Success: success when choosing the active action on the previous trial = 1, otherwise = 0), the effect of the difference between offers and costs presented on each trial (Offer - Cost), and the interaction between success on the last trial and the difference between offers and costs ((O - C) X Success).* ***b****) Shows the population-level posterior estimates of the second logistic regression model (Regression model 2) fitted separately in the different blocks and tasks. Beyond the Intercept the following population-level effects on choice (active = 1, passive = 0) were estimated: the effect of the number of trials with the same stimulus (Trial), the effect of the underlying success probability (Success Prob), and the interaction between trials with the same stimulus and probability to be successful if they chose the active action with that stimulus (Trial x Success Prob).*

Table S24. BF_{10} for regression model 1

	Robber 1	Factory 1	Robber 2	Factory 2
Success Last	2.59	**12.28**	0.32	0.33
Offer - Cost	**8.98e+14**	**4.28e+14**	**1.17e+14**	**1.27e+14**
Success x Offer-Cost	3.26	0.02	**638.84**	**8.06e+8**

Table S25. BF_{10} for regression model 2

	Robber 1	Factory 1	Robber 2	Factory 2
Trial	**60.75**	1.14	**7.18e+7**	**17.25**
Success Prob	0.45	0.34	0.36	0.5
Trial x Success Prob	2.91	0.42	**22.18**	**34.99**

Modeling results. To analyse the participant's choice data in the pilot study we fitted the same collection of choice models that are presented in the main manuscript. For each model comparison, we will present model frequency (*M.freq*), an estimate of the proportion of subjects in the sample that are best explained by a specific model and protected exceedance probability (*PXP*), indicating the probability that each model is the most likely across the group. All model fitting was carried out using Hierarchical Bayesian inference (HBI) from the computational and behavioral modeling (CBM)[2] toolbox in MATLAB (v 9.13.0.2105380 (R2022) Update 2).

In the first step, we analysed participant's choice data in the two tasks separately. Results show that for all blocks and tasks, except for the first block factory task, the Bayesian learner model had the highest model frequency. See Table S26 for full results. Furthermore, for all blocks and tasks, except for the first block of the factory task, the protected exceedance probability for the winning model was above 0.95 this indicates that this model is the most frequent in the sample.

Table S26. Model comparison for basic models.

Block	Task		SoftMax	Win-Stay Lose-Shift	Bayesian learner	Static Probability
Block 1	Robber	*M.freq*	0.1733	0.0893	**0.7374**	0.0000
		PXP	0.0000	0.0000	**1.0000**	0.0000
	Factory	*M.freq*	0.1152	0.2158	0.2794	**0.3896**
		PXP	0.0008	0.0362	0.1544	**0.8086**
Block 2	Robber	*M.freq*	0.1266	0.1196	**0.5404**	0.2133
		PXP	0.0001	0.0000	**0.9970**	0.0028
	Factory	*M.freq*	0.0727	0.1471	**0.4918**	0.2885
		PXP	0.0000	0.0005	**0.9530**	0.0465

Since the best-fitting model was the Bayesian learner model in at least one of the tasks (the robber task) in every block, we continued by testing the augmentations of the Bayesian model. Table S27 shows the result for each block when testing the basic version of the Bayesian learner models in head-to-head comparisons with the augmented versions of that model. Results show that in all model comparisons, the best-fitting model is the basic Bayesian model without augmentations.

Table S27. Model comparison for augmented Bayesian models.

Block	Task		Bayes vs. Continuous Updating		Bayes vs. Bidirectional Bias	
Block 1	Robber	*M.freq*	**0.8347**	0.1653	**0.7260**	0.2740
		PXP	**1.0000**	0.0000	**0.9996**	0.0004
	Factory	*M.freq*	**0.6421**	0.3579	**0.5215**	0.4785
		PXP	**0.9813**	0.0187	**0.6226**	0.3774
Block 2	Robber	*M.freq*	**0.6646**	0.3354	**0.5510**	0.4490
		PXP	**0.9921**	0.0079	**0.7712**	0.2288
	Factory	*M.freq*	**0.8027**	0.1973	**0.6660**	0.3340
		PXP	**1.0000**	0.0000	**0.9926**	0.0074

We next tested if the extracted parameters of the prior from the best fitting models correlated across the tasks. This provides an initial test of task invariance. The parameters were extracted from fitting the winning model to all participants. For the robber task and second block of the factory task this was the Bayesian learner, for the first block of the factory task this was the Static probability model. We then calculated Spearman's correlations between each of the parameters of the priors in the tasks. This was done separately for each block of the task. The results of the correlations between the parameters in the Robber and Factory task can be seen in Fig. S17. The p-values were calculated with 10 000 random permutations of the parameters of the prior to test the true correlation. Beyond these correlations, we also explored if other parameters of the models correlated, namely the temperature parameter (here, inverse temperature: *invT*). We found that in both blocks the *invT* parameter correlated across tasks tasks (Block 1: $r_s(52) = 0.438$, $p = 0.001$; Block 2: $r_s(52) = 0.719$, $p = 2.2e\text{-}16$).

To formally test task invariance, we next constructed two choice models based on the basic Bayesian learner model. One model assumed a shared prior across tasks and the other model assumed two separate priors, one for each task. Beyond this, the models also included a linear scaling parameter (ω) of the values of the factory task. Equation (7) shows how this parameter is implemented into the utility function of the factory task, effectively resulting in the two tasks having different *invT* parameters.

(7)

$$\text{Factory Task} \begin{cases} Q_{active,\, t,\, j} = -\, cost_{t,\, j} \cdot \omega - \left(\left(1 - subjP_{t,\, j}\right) \cdot offer_{t,\, j} \cdot \omega \right) \\ Q_{passive,\, t,\, j} = -\, offer_{t,\, j} \cdot \omega \end{cases}$$

We next performed a model comparison between these two models for each block separately. Since the results from these model comparisons differed between the first and second blocks of the task, we also performed a model comparison for the follow-up one week later. Results showed that for the second block and the follow up the 1-prior model was the best fit. For full results see Table S28.

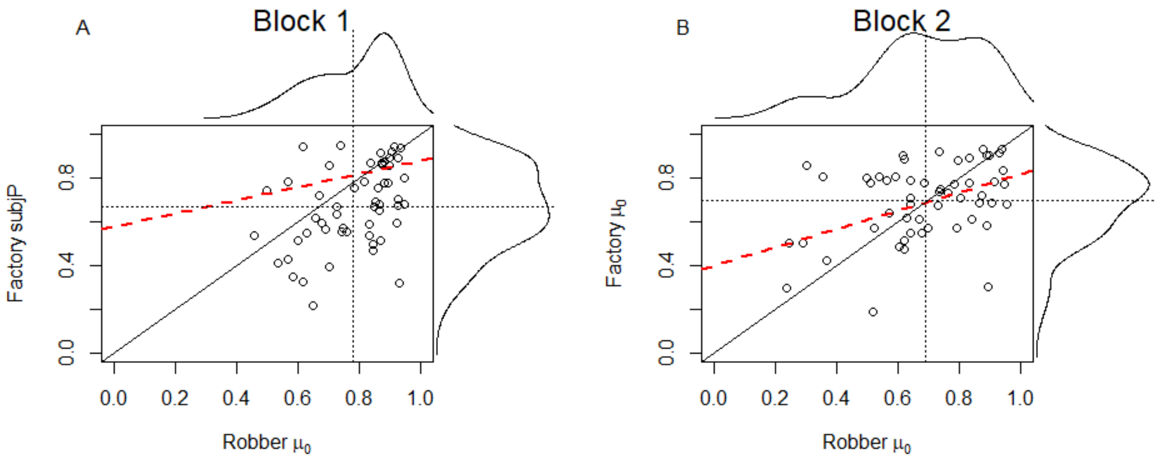

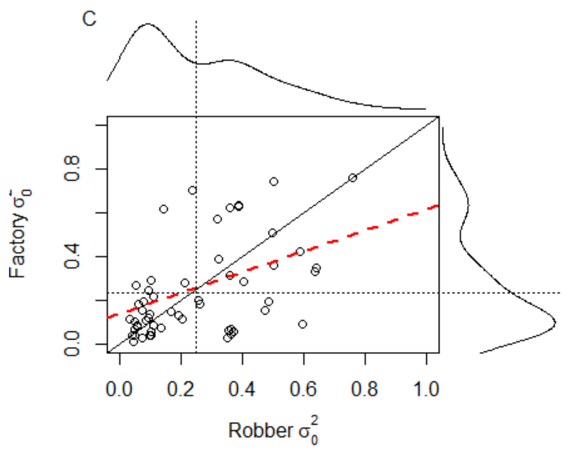

***Figure S17. Correlations between parameters of the prior across tasks.** Scatter plots show each participant's extracted parameters for each task and block. The dotted lines indicate the means of the parameters, and the diagonal indicates a perfect correlation. The red dashed lines indicate the regression lines. The figures above and to the right of the scatter plots show the distribution of the parameter values. Reported p-values from permutation test with 10 000 permutations. **a**) Figure shows the participants' parameter values for the mu and subjP parameters in the first block of the Robber and Factory task ($r_s(52)$ = 0.413, p = 7e-4). **b**) Figure shows the participants' parameter values for the mu parameter in the second block of the Robber and factory task ($r_s(52)$ = 0.314, p = 0.0124). **c**) Same as b but for the σ_0^2 parameter ($r_s(52)$ = 0.528, p = 3e-4).*

Table S28. Model comparison between 1- and 2-prior Bayesian models.

Session		1-Prior + ω	2-Prior + ω
Block 1	*M.freq*	0.4243	**0.5757**
	PXP	0.1349	**0.8651**
Block 2	*M.freq*	**0.5246**	0.4754
	PXP	**0.6399**	0.3601
Follow-Up	*M.freq*	**0.5798**	0.4202
	PXP	**0.8494**	0.1506

Test-retest reliability. To test the reliability of the parameters of the prior, participants performed the tasks in two blocks on the first day and the models were fitted separately for each block. We also invited all participants back to perform the tasks one week later again. Out of the 54 participants that performed the first day 43 participants (79.6 %) returned one week later. Reliability was measured in three different ways in the pilot study, explained variance (R^2), Spearman's correlation (r_s) and with two-way mixed effects, absolute agreement, single measure, inter-class correlations (ICC(2,1)). All measures were carried out in R. To calculate the ICC the R package *irr*[1] version 0.84.1 was used. Fig. S18a, d, g and j shows the within-session reliability and the other plots in Fig. S18 shows the reliability of the parameters between sessions and one week later, Table S29, S30 and S31 shows the reliability measures.

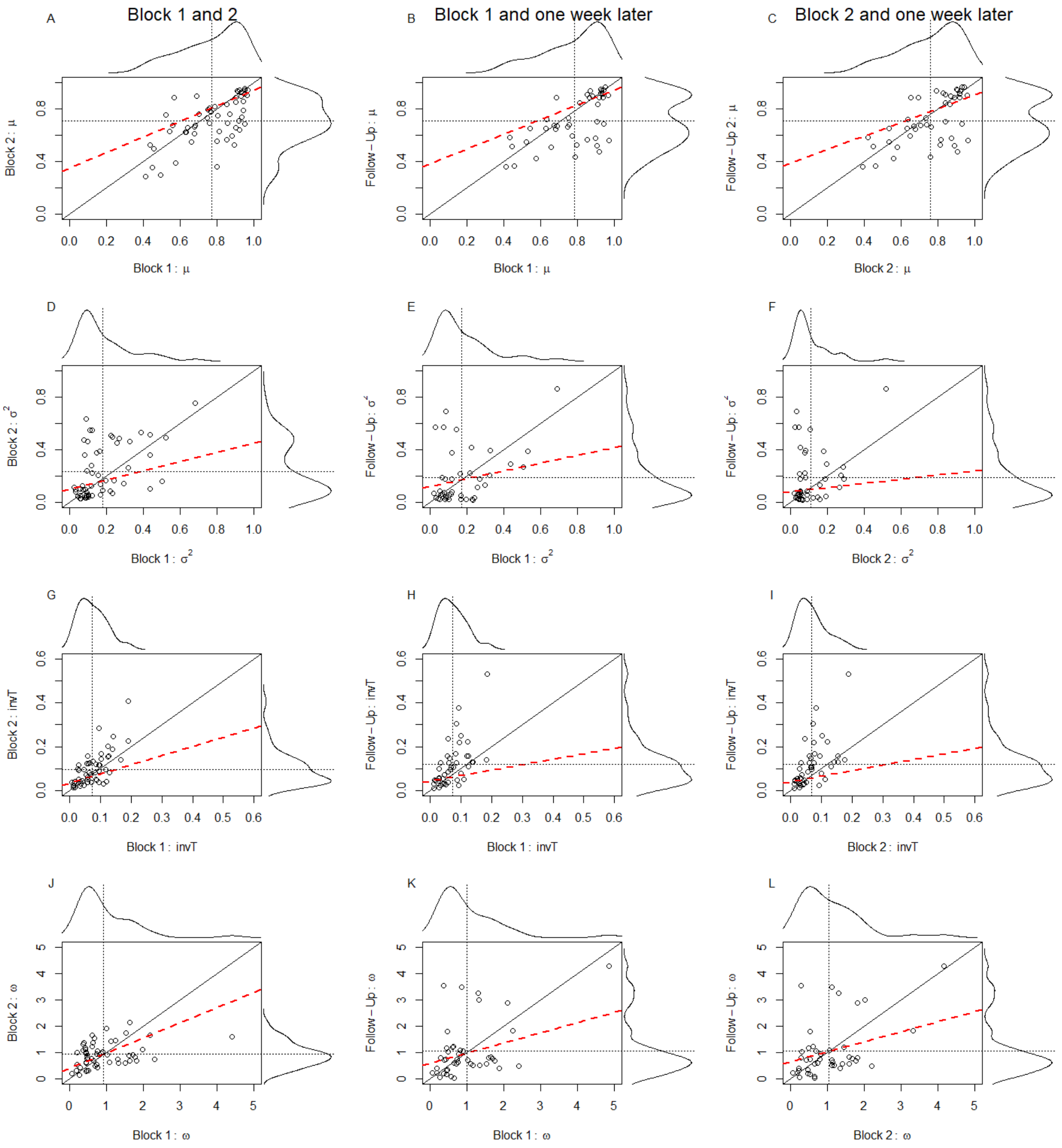

Figure S18. Test-retest reliability. *Each plot shows the estimated parameters of the same participant in at two different time points. Each point in each plot is the estimated parameter value for a participant, the dotted lines show the mean of the parameter at the different time points and the diagonal indicates a perfect correlation.* ***a****) Correlation between the task-invariant μ_0 parameter in blocks 1 and 2. Please note that N = 54.* ***b****) Correlation between the task-invariant μ_0 parameter in block 1 and the same parameter one week later. Please note that N = 43.* ***c)*** *Correlation between the task-invariant μ_0 parameter in block 2 and the same parameter one week later. Please note that N = 43.* ***d-f****) Same as a-c but for the σ_0^2 parameter.* ***g-i****) Same as a-c but for the invT parameter* ***j-l****) Same as a-c but for the ω parameter.*

Table S29. Test-retest reliability block 1 to block 2 to 1 week later.

Parameters	R^2	r_s	ICC(2,1)
Mu	0.220	0.639	0.602
Sigma	0.136	0.518	0.451
Inverse temperature	0.410	0.653	0.586
Omega	-1.448	0.287	0.320

Table S30. Test-retest reliability block 1

Parameters	R^2	r_s	ICC(2,1)
Mu	0.187	0.612	0.579
Sigma	0.121	0.253	0.406
Inverse temperature	0.155	0.636	0.370
Omega	0.139	0.355	0.472

Table S31. Test-retest reliability block 2 to one week later.

Parameters	R^2	r_s	ICC(2,1)
Mu	0.237	0.566	0.577
Sigma	-0.020	0.153	0.238
Inverse temperature	0.108	0.671	0.361
Omega	0.171	0.408	0.481

Correlations between parameters and questionnaires. To establish convergent validity for the priors, we used a large selection of questionnaires to explore which measures the priors may correlate with. Participants' estimated parameter values from the task invariant 1-prior Bayesian learner model were extracted from the model comparison between the 1- and 2-prior models. The parameters were transformed from the fitted parameters to the way they were applied in the model i.e., the Gaussian parameter values were transformed with a logistic function. Each participant's estimated μ_0 and σ_0^2 were then correlated with a Spearman's correlation (r_s) with the participant's total scores in each of the questionnaires. We also investigated if the questionnaires correlated with each other. Fig. S19 shows the correlations for the first block and Fig. S20 shows the correlations for the second block.

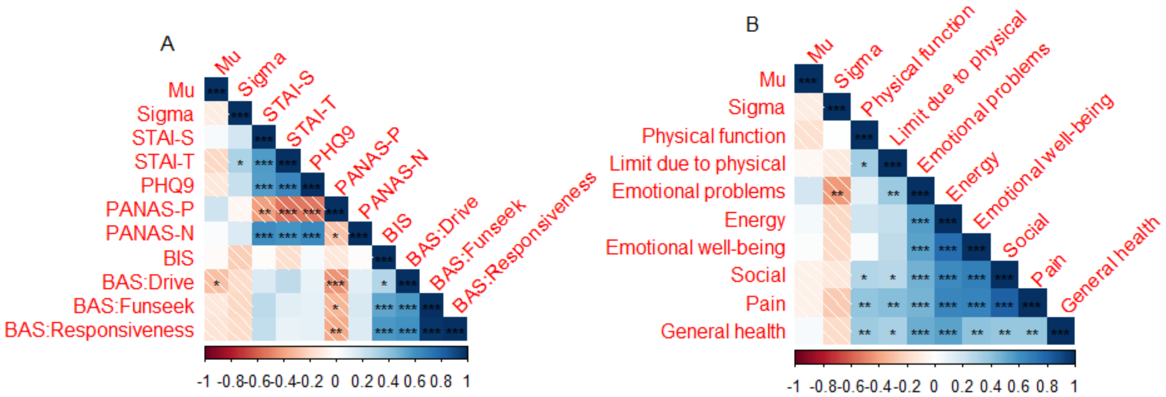

Figure S19. Correlations between parameters of the prior in block 1 and questionnaires. ** shows p < 0.05, ** shows p < 0.01, *** shows p < 0.001.* ***a****) Figure shows the correlation matrix for the following parameters of the prior extracted from the 1-prior model in the first block of the experiment and the following questionnaires: State-trait anxiety index (STAI), Patient health questionnaire 9 (PHQ9), Positive and negative affect schedule (PANAS), and the Behavioural inhibition and activation questionnaires (BIS/BAS). Each scale is split into its subscales. The STAI consist of state (S) and trait (T) subscales, where higher scores indicate higher levels of anxiety. We observe a significant correlation between the sigma parameter and STAI-T ($r_s(52) = 0.316$, $p = 0.0199$). The PANAS consist of Positive affect (P) and Negative affect (N), where higher scores indicate higher levels of affect. The BIS/BAS consist of 4 subscales: one Behavioural inhibition scale (BIS), and three behavioural activation scales (BAS). Higher scores here indicate more behavioural inhibition and lowered behavioural activation. We observe a significant correlation between the parameter mu and BAS drive subscale ($r_s(52) = -0.296$, $p = 0.03$).* ***b****) Figure shows the correlation matrix for the following parameters of the prior extracted from the 1-prior model in the first block of the experiment and the subscales of the RAND 36. Higher scores indicate better Physical function, less limitation due to physical functioning, fewer emotional problems, more energy, better emotional well-being, better social life, less pain and better general health. Here we found a correlation between the parameter sigma and Emotional problems ($r_s(52) = -0.4274$, $p = 0.0013$) indicating that with fewer emotional problems the participants' prior becomes more narrow.*

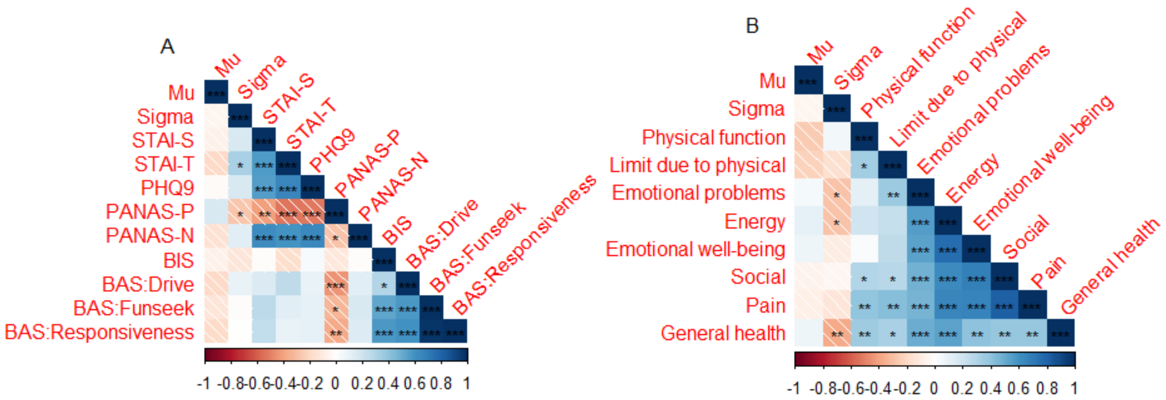

Figure S20. Correlations between parameters of the prior in block 2 and questionnaire scores. ** shows p < 0.05, ** shows p < 0.01, *** shows p < 0.001.* ***a****) Figure shows the correlation matrix for the following parameters of the prior extracted from the 1 prior model in the second block of the experiment and the following questionnaires: State-trait anxiety index (STAI), Patient health questionnaire 9 (PHQ9), Positive and negative affect schedule (PANAS), and the Behavioural inhibition and activation questionnaires (BIS/BAS). Each scale is split into its subscales. The STAI consist of state (S) and trait (T) subscales, where higher scores indicate higher levels of anxiety. We observe a significant correlation between the sigma parameter and STAI-T ($r_s(52) = 0.328$, $p = 0.0155$). The PANAS consist of Positive affect (P) and Negative affect (N), where higher scores indicate higher levels of affect. Here we see a significant correlation between the sigma parameter and PANAS-P($r_s(52) = -0.28$, $p = 0.0404$). The BIS/BAS consist of 4 subscales: one Behavioural inhibition scale (BIS), and three behavioural activation scales (BAS). Higher scores here indicate more apathy, more behavioural inhibition and lower behavioural activation.* ***b)*** *Figure shows the correlation matrix for the following parameters of the prior extracted from the 1 prior model in the second block of the experiment and the subscales of the RAND 36. Higher scores indicate better Physical function, less limitation due to physical functioning, fewer emotional problems, more energy, better emotional well-being, better social life, less pain and better general health. Here we found a correlation between the parameter sigma and Emotional problems ($r_s(52) = -0.2865$, $p = 0.0357$), Energy ($r_s(52) = -0.2879$, $p = 0.0348$) and general health ($r_s(52) = -0.375$, $p = 0.0052$)*

As a result of our hypotheses and these results, we decided to focus the main study on positive affect as measured by the PANAS and non-specific negative affect as measured by the STAI. Beyond our hypothesis about the impact of a pessimistic prior on positive affect, the PANAS-P also has clear strong correlations with depressive symptoms as measured with the PHQ9 ($r_s(52) = -0.5295$, $p = 0$) and behavioural activation as measured with the BIS/BAS (Drive: $r_s(52) = -0.4438$, p = 8^{-4}; Funseeking: $r_s(52) = -0.3319$, $p = 0.0142$; Responsiveness: $r_s(52) = -0.3759$, $p = 0.0051$), making it a central measure in our study. However, in this smaller sample, we do not see significant correlations between the mu parameter and positive affect as measured by the PANAS-P (Block 1: $r_s(52) = 0.2072$, $p = 0.1328$; Block 2: $r_s(52) = 0.1691$, $p = 0.2216$). What we do find consistently across blocks is a correlation between STAI-T and the sigma parameter (Block 1: $r_s(52) = 0.316$, $p = 0.0199$; Block 2: $r_s(52) = 0.328$, $p = 0.0155$).

Supplementary References

1. Gamer, M., Lemon, J., Fellows, I. & Singh, P. irr: Various Coefficients of Interrater Reliability and Agreement. (2019).
2. Piray, P., Dezfouli, A., Heskes, T., Frank, M. J. & Daw, N. D. Hierarchical Bayesian inference for concurrent model fitting and comparison for group studies. *PLoS Comput Biol* **15**, e1007043 (2019).
3. Watson, D., Clark, L. A. & Tellegen, A. Development and Validation of Brief Measures of Positive and Negative Affect: The PANAS Scales. *Journal of Personality and Social Psychology* **54**, 1063–1070 (1988).
4. Spielberger, C. D., Gorsuch, R. L., Lushene, R., Vagg, P. R. & Jacobs, G. A. *State-Trait Anxiety Inventory for Adults Sampler Set Manual, Instrument and Scoring Guide*. (1983).
5. Kroenke, K., Spitzer, R. L. & Williams, J. B. W. The PHQ-9: Validity of a brief depression severity measure. *J Gen Intern Med* **16**, 606–613 (2001).
6. Radloff, L. S. The CES-D Scale: A Self-Report Depression Scale for Research in the General Population. *Applied Psychological Measurement* **1**, 385–401 (1977).
7. Rush, J. A. *et al.* The inventory for depressive symptomatology (IDS): Preliminary findings. *Psychiatry Research* **18**, 65–87 (1986).
8. Zung, W. W. K. A Self-Rating Depression Scale. *Arch Gen Psychiatry* **12**, 63 (1965).
9. Molina, S. & Borkovec, T. D. The Penn State Worry Questionnaire: Psychometric properties and associated characteristics. in *Worrying: Perspectives on theory, assessment and treatment* 265–283 (John Wiley & Sons, Oxford, England, 1994).
10. Spitzer, R. L., Kroenke, K., Williams, J. B. W. & Löwe, B. A Brief Measure for Assessing Generalized Anxiety Disorder: The GAD-7. *Archives of Internal Medicine* **166**, 1092–1097 (2006).
11. Carver, C. S. & White, T. L. Behavioral inhibition, behavioral activation, and affective responses to impending reward and punishment: The BIS/BAS Scales. *Journal of Personality and Social Psychology* **67**, 319–333 (1994).

12. Ang, Y.-S., Lockwood, P., Apps, M. A. J., Muhammed, K. & Husain, M. Distinct Subtypes of Apathy Revealed by the Apathy Motivation Index. *PLOS ONE* **12**, e0169938 (2017).
13. Snaith, R. P. *et al.* A scale for the assessment of hedonic tone: The Snaith-Hamilton pleasure Scale. *British journal of psychiatry* **167**, 99–103 (1995).
14. Ware, J. E. & Sherbourne, C. The MOS 36-ltem Short-Form Health Survey (SF-36): I. Conceptual Framework and Item Selection. *Med Care* **30**, 473–483 (1992).
15. Bürkner, P.-C. Advanced Bayesian Multilevel Modeling with the R Package brms. *The R Journal* **10**, 395–411 (2018).
16. Bürkner, P.-C. brms : An R Package for Bayesian Multilevel Models Using Stan. *J. Stat. Soft.* **80**, 1–28 (2017).
17. Mcelreath, R. *Statistical Rethinking A Bayesian Course with Examples in R and Stan*. (2015).
18. Lewandowski, D., Kurowicka, D. & Joe, H. Generating random correlation matrices based on vines and extended onion method. *Journal of Multivariate Analysis* **100**, 1989–2001 (2009).
